# Supplementary material for: Targeted delivery of celastrol to mesangial cells is effective against mesangioproliferative glomerulonephritis
Source: Nat Commun. 2017 Oct 12;8:878. doi: 10.1038/s41467-017-00834-8 (PMC5638829; doi:10.1038/s41467-017-00834-8)
Supplement: Supplementary file 1 — Supplementary Information [file 41467_2017_834_MOESM1_ESM.pdf]

Description of Supplementary Files

File name: Supplementary Information
Description: Supplementary figures and supplementary tables.

File name: Peer review file

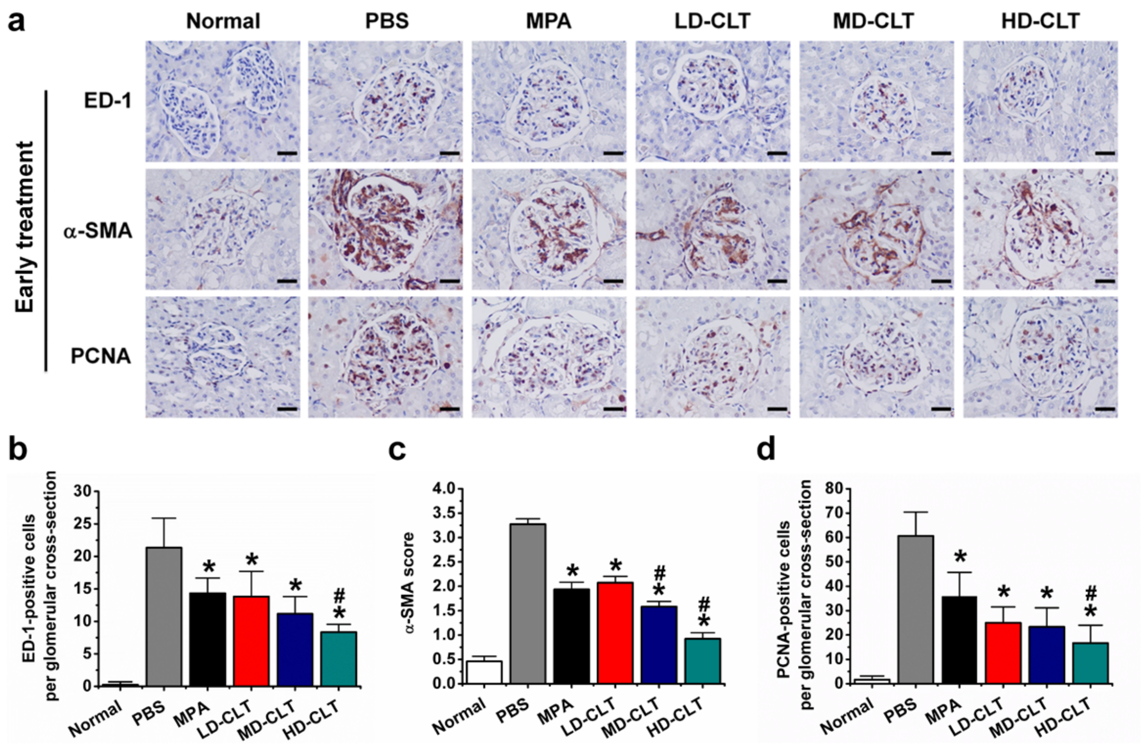

Supplementary Figure 1. Therapeutic efficacy of early CLT treatment against macrophage infiltration, mesangial cells activation and proliferation in the reversible anti-Thy1.1 nephritis model. (a) Representative photomicrographs of immunostaining for ED-1, α-SMA and PCNA in kidney tissue sections from anti-Thy1.1 nephritic rats after early treatment with MPA (30 mg kg^{-1}) and CLT (LD-CLT, 1 mg kg^{-1}; MD-CLT, 2 mg kg^{-1}; HD-CLT, 3 mg kg^{-1}). Scale bars, 20 μm. **(b)** Effects of MPA and different doses of CLT on macrophage infiltration on day 1 after disease induction. For each animal group, 150 glomeruli were selected and ED-1 positive cells were counted using cellSens Standard digital imaging software (Olympus). **(c)** Effects of MPA and different doses of CLT on mesangial cell activation on day 5 after disease induction. For each animal group, 150 glomeruli were analyzed and α-SMA staining was graded semiquantitatively as described in Methods. **(d)** Effects of MPA and different doses of CLT on mesangial cell proliferation on day 5 after disease induction. For each animal group, 150 glomeruli were selected and PCNA-positive cells were counted using cellSens Standard digital imaging software (Olympus). In panels **(b)** to **(d)**, data are mean ± s.d. (n = 5), results are representative of two independent experiments. $^{*}P < 0.05$ *versus* PBS group; $^{\#}P < 0.05$ *versus* MPA group. Statistical significance was determined by one-way ANOVA with Tukey *post hoc* test.

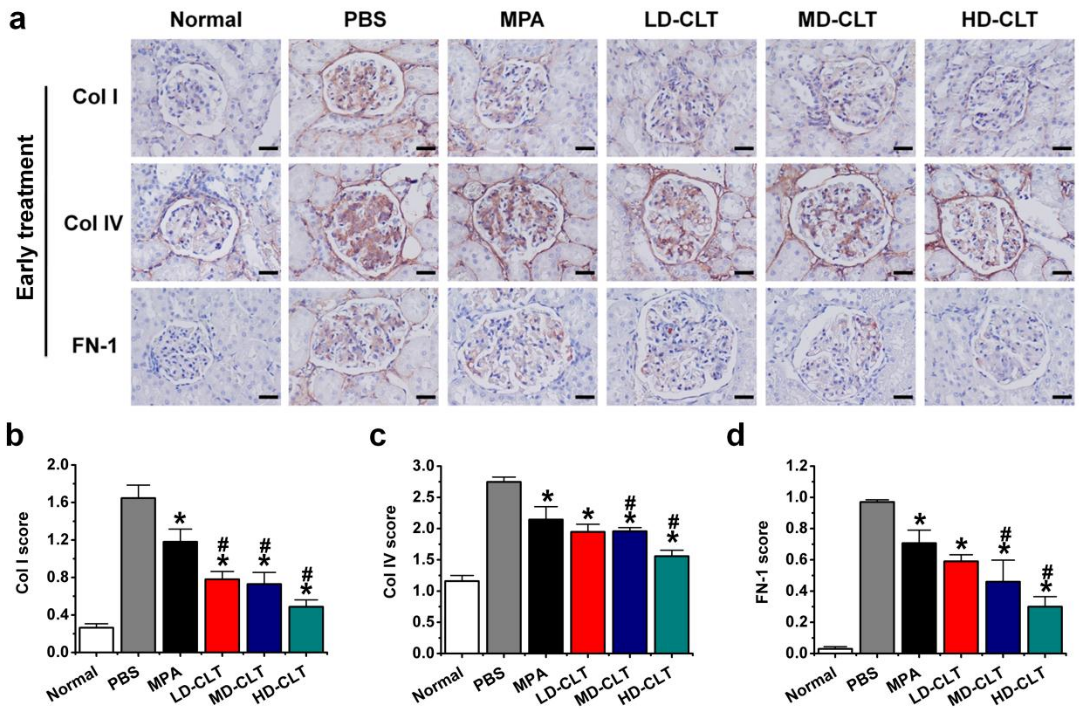

Supplementary Figure 2. The inhibitory effect of early therapy of CLT on the deposition of ECM protein in the reversible anti-Thy1.1 nephritis model. (a) Representative photomicrographs of immunostaining for Col I, Col IV and FN-1 in kidney tissue sections from anti-Thy1.1 nephritic rats after early treatment with MPA (30 mg kg^{-1}) and CLT (LD-CLT, 1 mg kg^{-1}; MD-CLT, 2 mg kg^{-1}; HD-CLT, 3 mg kg^{-1}). Scale bars, 20 μm. **(b-d)** Effects of MPA and different doses of CLT on deposition of Col I **(b)**, Col IV **(c)** and FN-1 **(d)** on day 5 after disease induction. For each animal group, 150 glomeruli were analyzed and staining of ECM proteins was graded semiquantitatively as described in Methods. In panels **(b)** to **(d),** data are mean ± s.d. (n = 5), results are representative of two independent experiments. $^{*}P < 0.05$ *versus* PBS group; $^{\#}P < 0.05$ *versus* MPA group. Statistical significance was determined by one-way ANOVA with Tukey *post hoc* test.

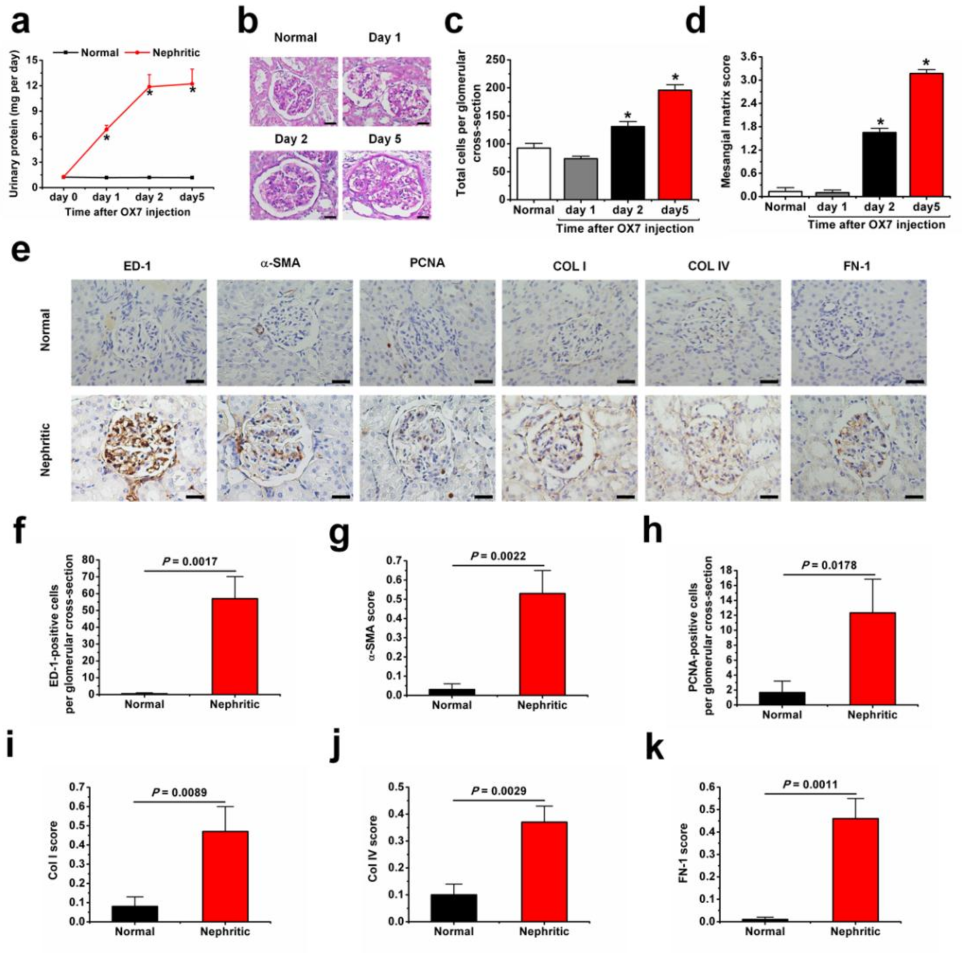

Supplementary Figure 3. Rats shows marked proteinuria, macrophage infiltration, glomerular hypercellularity and ECM accumulation at day 2 after disease induction. **(a)** 24-h urinary protein excretion in rats after disease induction. Data are mean ± s.d. (n = 5), $^*P < 0.05$ *versus* normal group, statistical significance was determined using the Student's t-test. **(b)** Glomerular histology revealed by PAS staining of kidney tissue sections from rats after disease induction. Scale bars, 20μm. **(c)** The total glomerular cellularity in rats after disease induction. **(d)** The ECM deposition in rats after disease induction. In panels **(c)** and **(d)**, data are mean ± s.d. (n = 5), $^*P < 0.05$ *versus* normal group, statistical significance was determined using the Student's t-test. **(e)** Representative photomicrographs of immunostaining for ED-1, α-SMA, PCNA, Col I, Col IV and FN-1 in kidney tissue sections from rats at day 2 after disease induction. Scale bars, 20 μm. **(f-k)** The immunochemical results of ED-1 **(f)**, α-SMA **(g)**, PCNA **(h)**, Col I **(i)**, Col IV **(j)** and FN-1 **(k)** were semiquantitatively evaluated as described in Methods. Data are mean ± s.d. (n = 5). P values were calculated by Student's t-test.

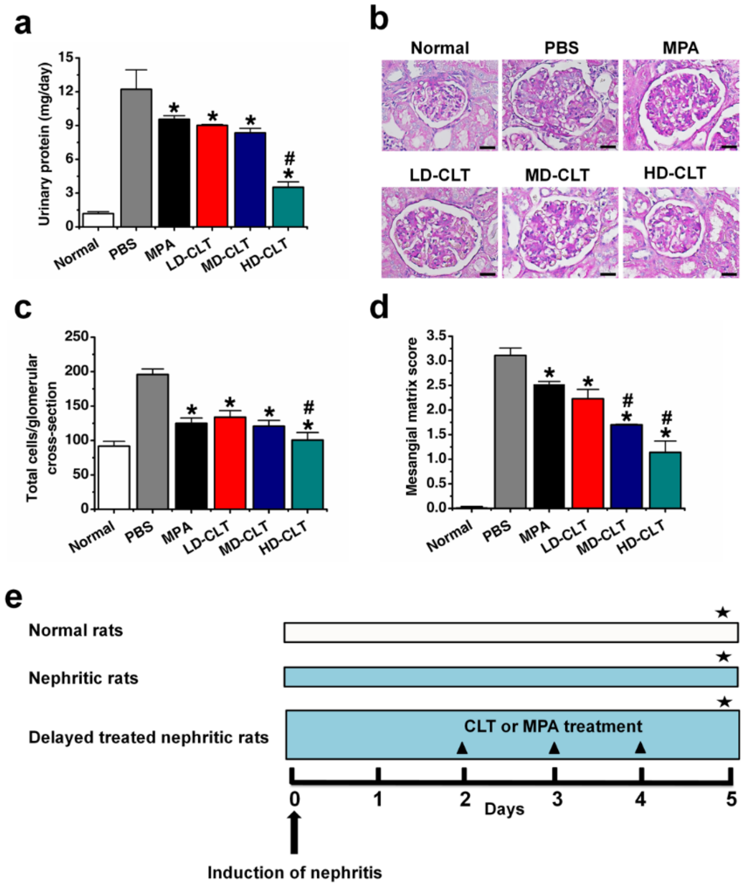

Supplementary Figure 4. Delayed therapy of CLT shows dose-dependent therapeutic efficacy in the reversible anti-Thy1.1 nephritis model. (a) Effects of delayed MPA (30 mg kg^{-1}) and CLT treatment (LD-CLT, 1 mg kg^{-1}; MD-CLT, 2 mg kg^{-1}; HD-CLT, 3 mg kg^{-1}) on 24-h urinary protein excretion in anti-Thy1.1 nephritic rats on day 5 after disease induction. **(b)** Glomerular histology revealed by PAS staining of kidney tissue sections from anti-Thy1.1 nephritic rats on day 5 after delayed treatment with MPA or different doses of CLT. Scale bars, 20μm. **(c)** Effects of MPA and different doses of CLT on total glomerular cellularity on day 5 after disease induction. For each animal group, 150 glomeruli were selected and total glomerular cells were counted using cellSens Standard digital imaging software (Olympus). **(d)** Effects of MPA and different doses of CLT on ECM accumulation on day 5 after disease induction. For each animal group, 150 glomeruli were analyzed and ECM deposition was graded semiquantitatively as described in Methods. In panels **(a)**, **(c)** and **(d)**, data are mean ± s.d. (n = 5), results are representative of two independent experiments. $^{*}P < 0.05$ *versus* PBS group; $^{\#}P < 0.05$ *versus* MPA group. Statistical significance was determined by one-way ANOVA with Tukey *post hoc* test. **(e)** Flow diagram of the delayed treatment of CLT or MPA against the reversible anti-Thy1.1 nephritis. Black triangle denotes intravenous treatment of MPA or CLT; black star denotes time points of nephrectomy while respective animals were sacrificed. A detailed description is given in Methods.

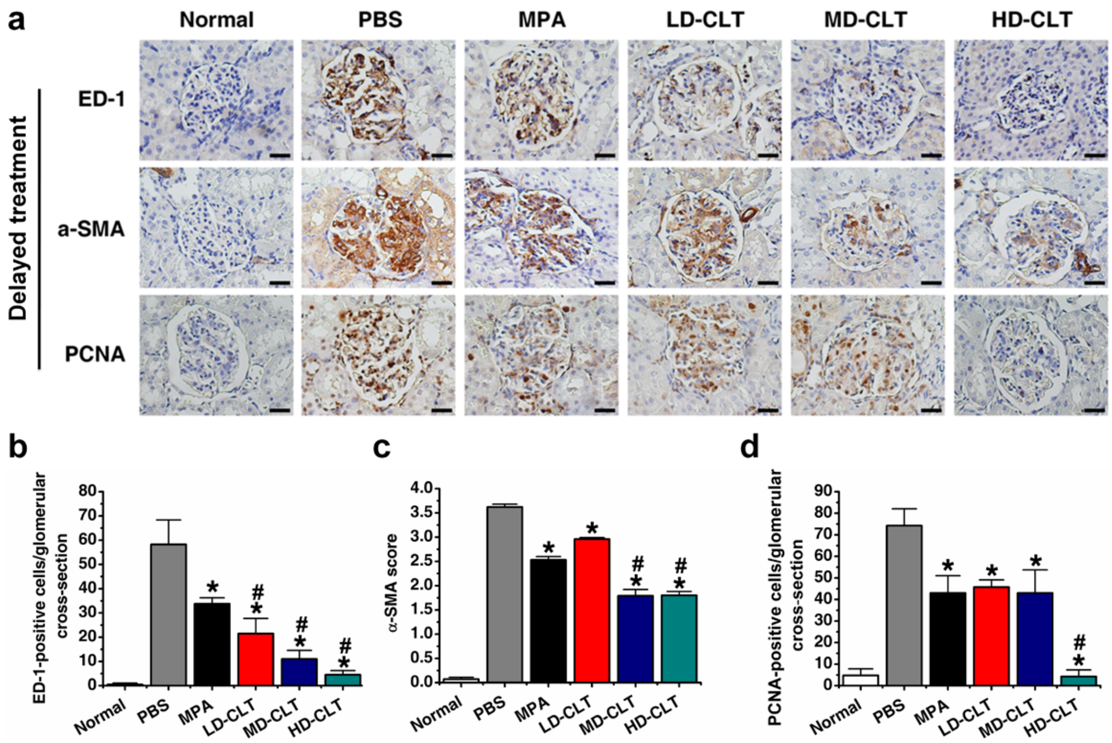

Supplementary Figure 5. Therapeutic efficacy of delayed treatment of CLT against macrophage infiltration, mesangial cells activation and proliferation in the reversible anti-Thy1.1 nephritis model. (a) Representative photomicrographs of immunostaining for ED-1, α-SMA and PCNA in kidney tissue sections from anti-Thy1.1 nephritic rats after delayed treatment with MPA (30 mg kg^{-1}) and CLT (LD-CLT, 1 mg kg^{-1}; MD-CLT, 2 mg kg^{-1}; HD-CLT, 3 mg kg^{-1}). Scale bars, 20 μm. **(b)** Effects of MPA and different doses of CLT on macrophage infiltration on day 5 after disease induction. For each animal group, 150 glomeruli were selected and ED-1 positive cells were counted using cellSens Standard digital imaging software (Olympus). **(c)** Effects of MPA and different doses of CLT on mesangial cell activation on day 5 after disease induction. For each animal group, 150 glomeruli were analyzed and α-SMA staining was graded semiquantitatively as described in Methods. **(d)** Effects of MPA and different doses of CLT on mesangial cell proliferation on day 5 after disease induction. For each animal group, 150 glomeruli were selected and PCNA-positive cells were counted using cellSens Standard digital imaging software (Olympus). In panels **(b)** to **(d)**, data are mean ± s.d. (n = 5), results are representative of two independent experiments. $^{*}P < 0.05$ *versus* PBS group; $^{\#}P < 0.05$ *versus* MPA group. Statistical significance was determined by one-way ANOVA with Tukey *post hoc* test.

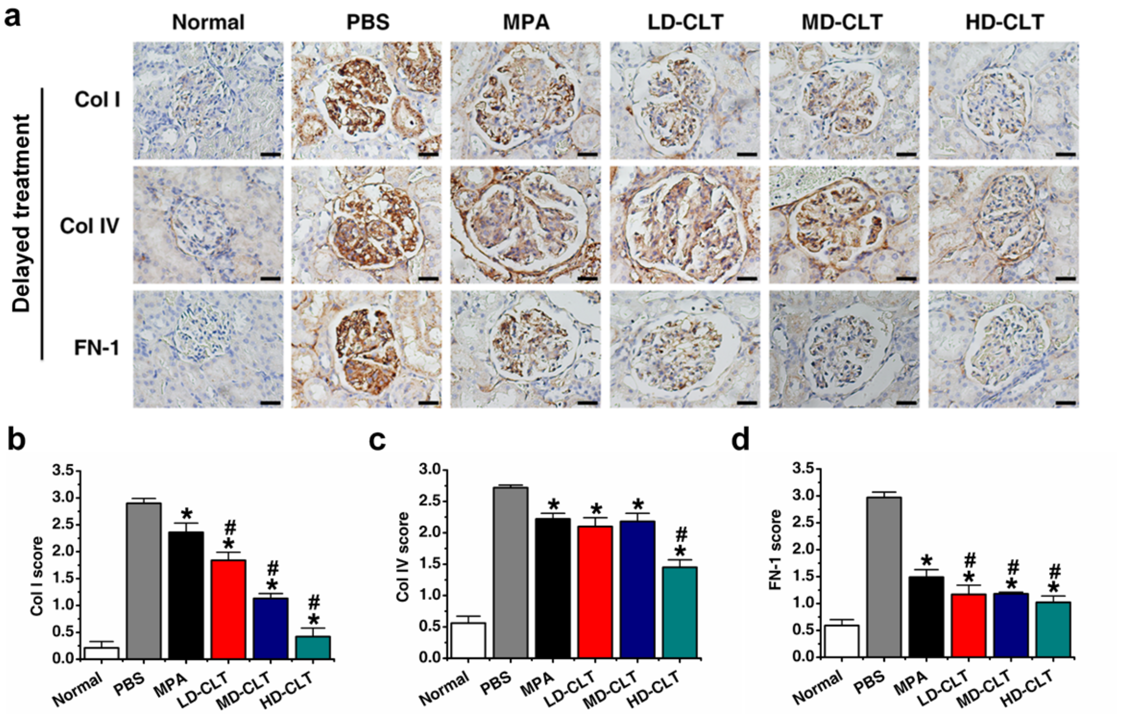

Supplementary Figure 6. The inhibitory effect of delayed therapy of CLT on the deposition of ECM protein in the reversible anti-Thy1.1 nephritis model. (a) Representative photomicrographs of immunostaining for Col I, Col IV and FN-1 in kidney tissue sections from anti-Thy1.1 nephritic rats after delayed treatment with MPA (30 mg kg^{-1}) and CLT (LD-CLT, 1 mg kg^{-1}; MD-CLT, 2 mg kg^{-1}; HD-CLT, 3 mg kg^{-1}). Scale bars, 20 μm. **(b-d)** Effects of MPA and different doses of CLT on deposition of Col I **(b)**, Col IV **(c)** and FN-1 **(d)** on day 5 after disease induction. For each animal group, 150 glomeruli were analyzed and staining of ECM proteins was graded semiquantitatively as described in Methods. In panels **(b)** to **(d)**, data are mean ± s.d. (n = 5), results are representative of two independent experiments. $^{*}P < 0.05$ *versus* PBS group; $^{\#}P < 0.05$ *versus* MPA group. Statistical significance was determined by one-way ANOVA with Tukey *post hoc* test.

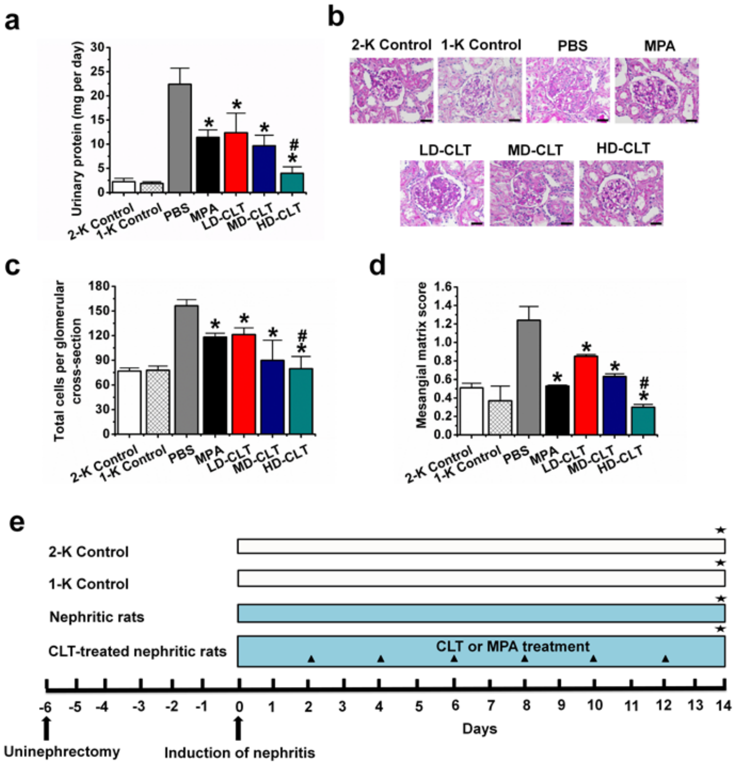

Supplementary Figure 7. Delayed CLT treatment shows dose-dependent therapeutic efficacy in the irreversible anti-Thy1.1 nephritis model. (a) Effects of MPA (30 mg kg^{-1}) and CLT treatment (LD-CLT, 1 mg kg^{-1}; MD-CLT, 2 mg kg^{-1}; HD-CLT, 3 mg kg^{-1}) on 24-h urinary protein excretion in anti-Thy1.1 nephritic rats on day 14 after disease induction. **(b)** Glomerular histology revealed by PAS staining of kidney tissue sections from anti-Thy1.1 nephritic rats on day 14 after delayed treatment with MPA or different doses of CLT. Scale bars, 20μm. **(c)** Effects of MPA and different doses of CLT on total glomerular cellularity on day 14 after disease induction. For each animal group, 150 glomeruli were selected and total glomerular cells were counted using cellSens Standard digital imaging software (Olympus). **(d)** Effects of MPA and different doses of CLT on ECM accumulation on day 14 after disease induction. For each animal group, 150 glomeruli were analyzed and ECM deposition was graded semiquantitatively as described in Methods. In panels **(a)**, **(c)** and **(d)**, data are mean ± s.d. (n = 5), results are representative of two independent experiments. Nonnephrectomized two-kidney control (2-K Control) and uninephrectomized one-kidney controls (1-K Control) served as controls. $^{*}P < 0.05$ *versus* PBS group; $^{\#}P < 0.05$ *versus* MPA group. Statistical significance was determined by one-way ANOVA with Tukey *post hoc* test. **(e)** Flow diagram of the CLT or MPA treatment against the irreversible anti-Thy1.1 nephritis. black triangle denotes intravenous treatment of CLT or MPA; black star denotes time points of nephrectomy while respective animals were sacrificed. A detailed description is given in Methods.

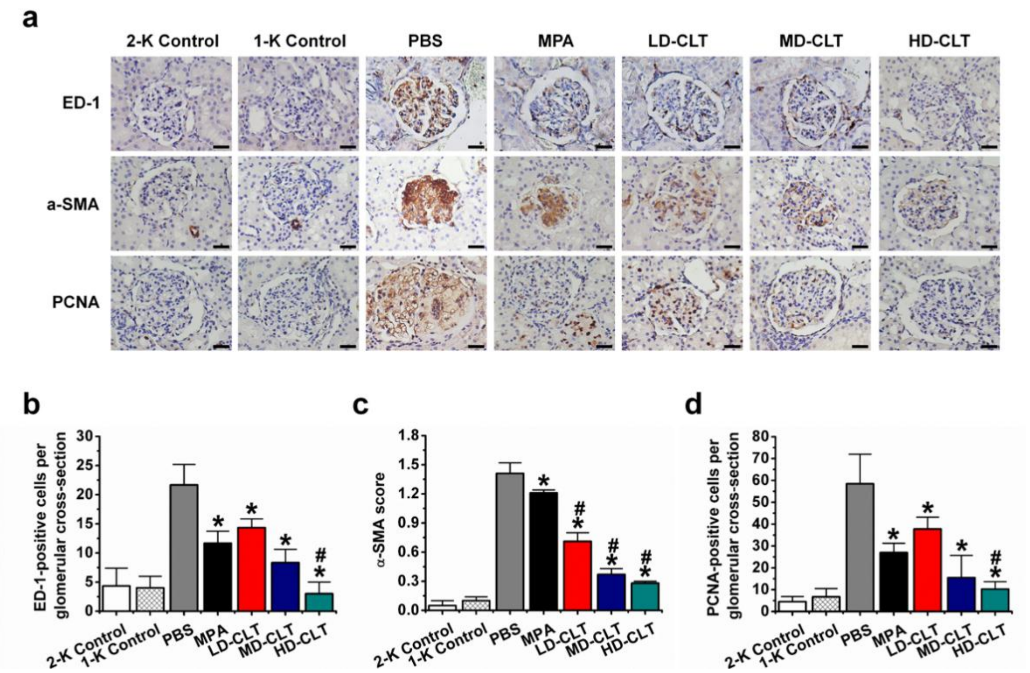

Supplementary Figure 8. Therapeutic efficacy of delayed CLT treatment against macrophage infiltration, mesangial cells activation and proliferation in the irreversible anti-Thy1.1 nephritis model. (a) Representative photomicrographs of immunostaining for ED-1, α-SMA and PCNA in kidney tissue sections from anti-Thy1.1 nephritic rats after delayed treatment with MPA (30 mg kg^{-1}) and CLT (LD-CLT, 1 mg kg^{-1}; MD-CLT, 2 mg kg^{-1}; HD-CLT, 3 mg kg^{-1}). Scale bars, 20 μm. **(b)** Effects of MPA and different doses of CLT on macrophage infiltration on day 14 after disease induction. For each animal group, 150 glomeruli were selected and ED-1 positive cells were counted using cellSens Standard digital imaging software (Olympus). **(c)** Effects of MPA and different doses of CLT on mesangial cell activation on day 14 after disease induction. For each animal group, 150 glomeruli were analyzed and α-SMA staining was graded semiquantitatively as described in Methods. **(d)** Effects of MPA and different doses of CLT on mesangial cell proliferation on day 14 after disease induction. For each animal group, 150 glomeruli were selected and PCNA-positive cells were counted using cellSens Standard digital imaging software (Olympus). In panels **(b)** to **(d)**, data are mean ± s.d. (n = 5), results are representative of two independent experiments. Nonnephrectomized two-kidney control (2-K Control) and uninephrectomized one-kidney control (1-K Control) served as controls. $^{*}P < 0.05$ *versus* PBS group; $^{\#}P < 0.05$ *versus* MPA group. Statistical significance was determined by one-way ANOVA with Tukey *post hoc* test.

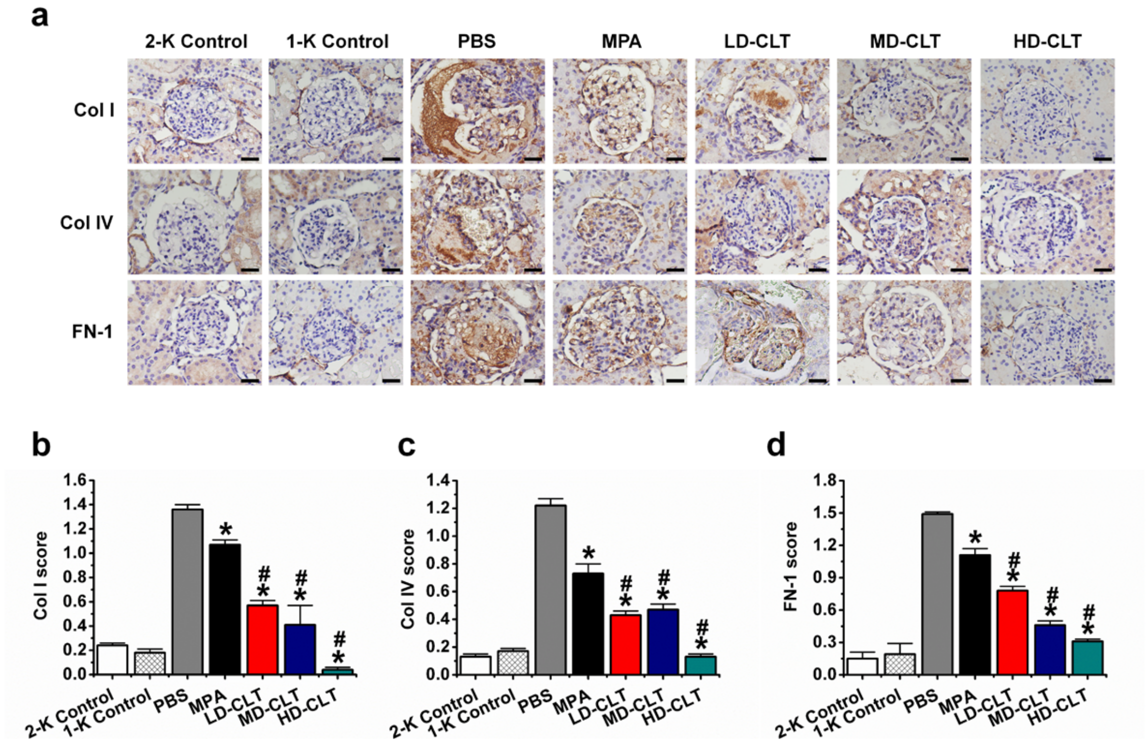

Supplementary Figure 9. The inhibitory effect of delayed CLT treatment on the deposition of ECM protein in the irreversible anti-Thy1.1 nephritis model. (a) Representative photomicrographs of immunostaining for Col I, Col IV and FN-1 in kidney tissue sections from anti-Thy1.1 nephritic rats after delayed treatment with MPA (30 mg kg^{-1}) and CLT (LD-CLT, 1 mg kg^{-1}; MD-CLT, 2 mg kg^{-1}; HD-CLT, 3 mg kg^{-1}). Scale bars, 20 μm. **(b-d)** Effects of MPA and different doses of CLT on deposition of Col I **(b)**, Col IV **(c)** and FN-1 **(d)** on day 14 after disease induction. For each animal group, 150 glomeruli were analyzed and staining of ECM proteins was graded semiquantitatively as described in Methods. In panels **(b)** to **(d)**, data are mean ± s.d. (n = 5), results are representative of two independent experiments. Nonnephrectomized two-kidney control (2-K Control) and uninephrectomized one-kidney control (1-K Control) served as controls. $^{*}P < 0.05$ *versus* PBS group; $^{\#}P < 0.05$ *versus* MPA group. Statistical significance was determined by one-way ANOVA with Tukey *post hoc* test.

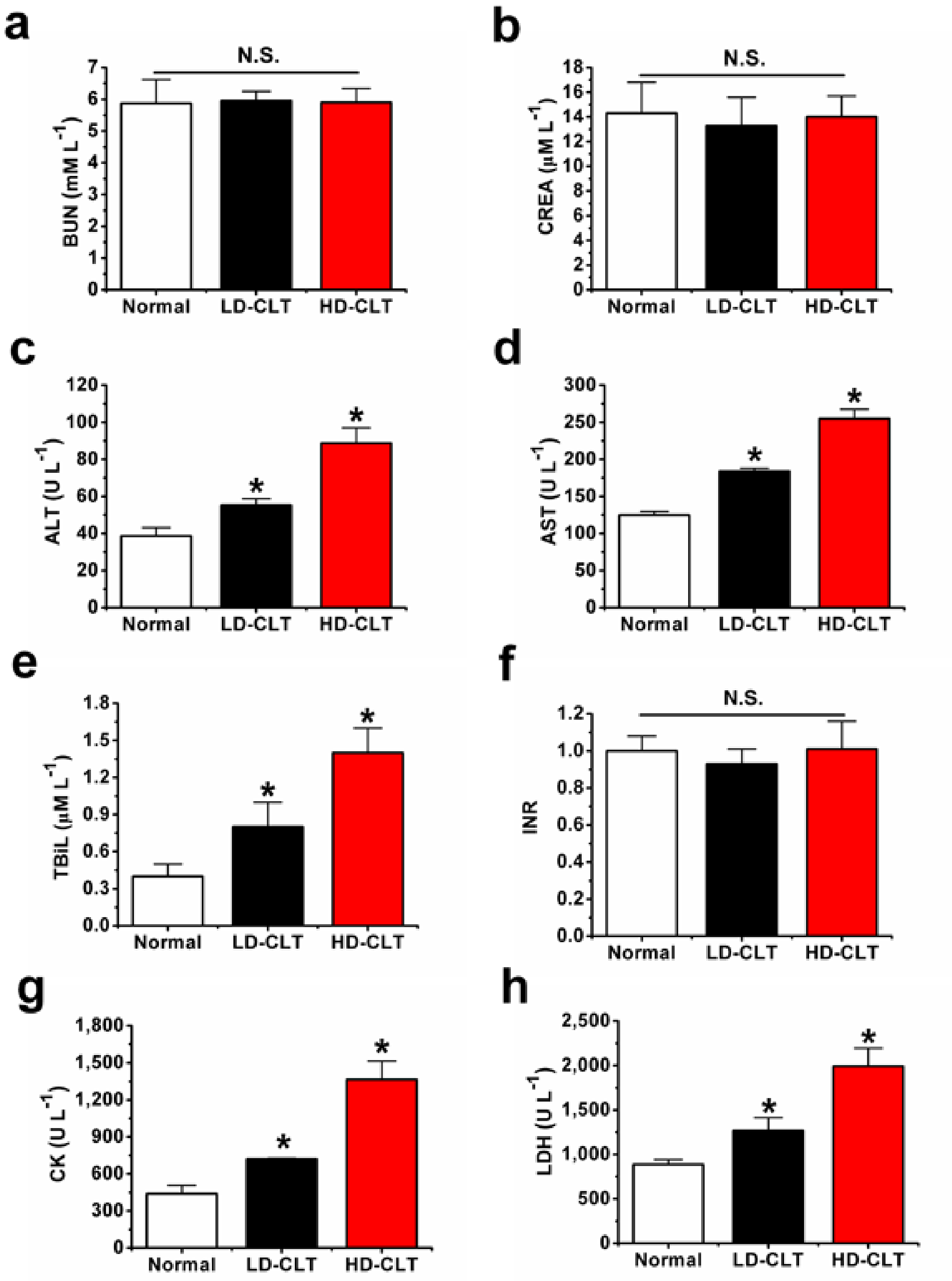

Supplementary Figure 10. The levels of biochemical parameters after intravenous treatment with saline or CLT (LD-CLT, 1 mg kg^{-1}; HD-CLT, 3 mg kg^{-1}) for 5 days. BUN **(a)** and CREA **(b)** were determined for evaluating the renal function. ALT **(c)**, AST **(d)**, TBiL **(e)** and INR **(f)** were measured for reflecting the liver injury or dysfunction. CK **(g)** and LDH **(h)** were measured for reflecting the cardiac injury. Data are mean ± s.d. (n = 5), results are representative of two independent experiments. N.S., not significant, $^{*}P < 0.05$ *versus* normal group. Statistical significance was determined using the Student's *t*-test.

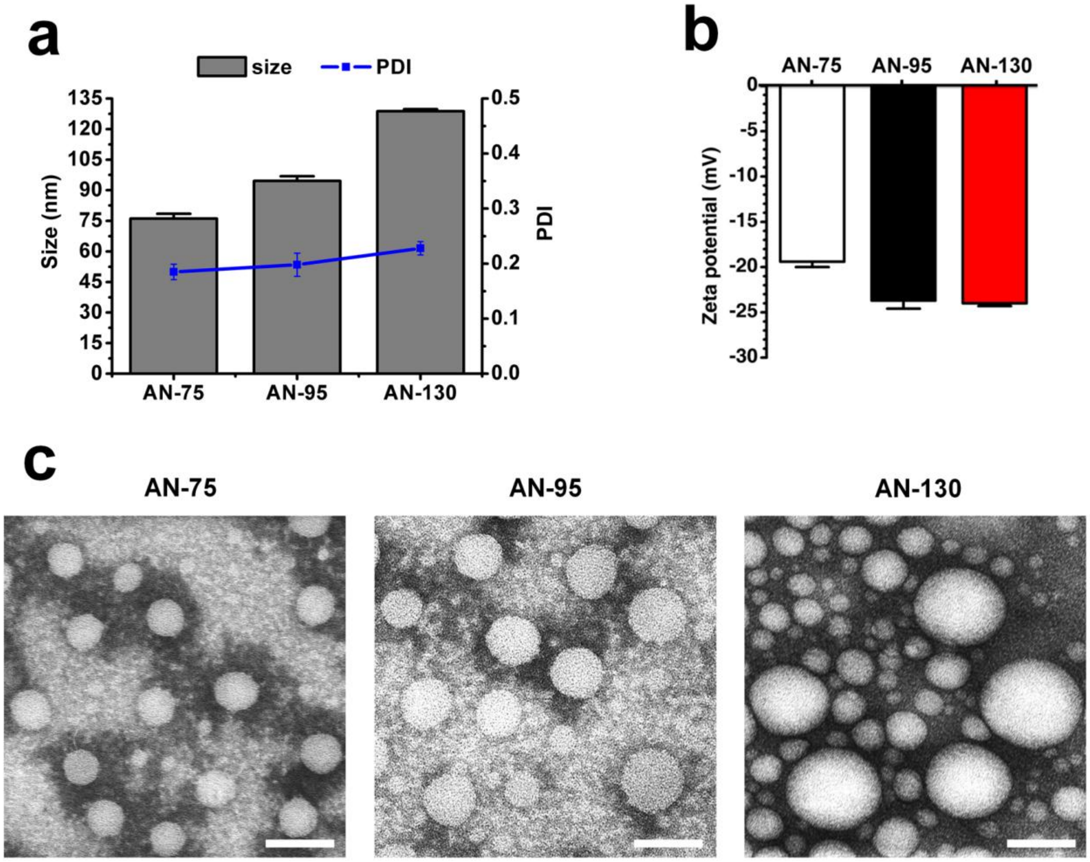

Supplementary Figure 11. Characterizations of FITC-ANs. (a) Size and polydispersity index (PDI) of FITC-ANs, as determined by dynamic light scattering. **(b)** Zeta potential of FITC-ANs. **(c)** Transmission electron micrographs of FITC-ANs. Scale bars, 100 nm. In panels **(a)** and **(b)**, data are mean ± s.d. (n = 3), results are representative of three independent experiments.

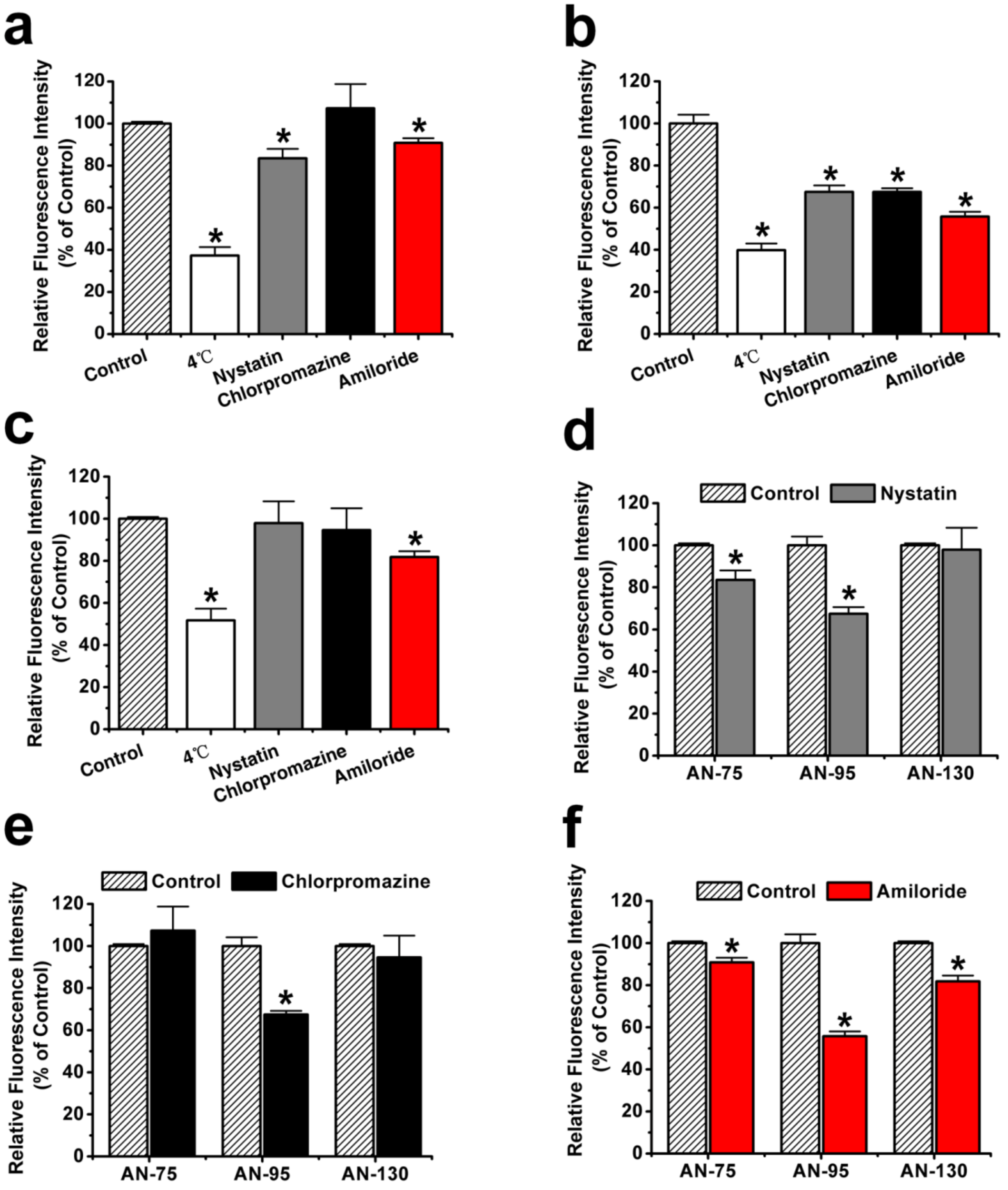

Supplementary Figure 12. Effects of 4 ℃ and endocytic inhibitors on cellular uptake of FITC-ANs by mesangial cells. (a-c) Cellular uptake of AN-75 **(a)**, AN-95 **(b)** and AN-130 **(c)** by mesangial cells. **(d-f)** Effects of nystatin **(d)**, chlorpromazine **(e)** and amiloride **(f)** on cellular uptake of FITC-ANs by mesangial cells. The groups in the presence of tested FITC-ANs but without inhibitor treatments were used as controls and their fluorescence intensities were expressed as 100%. Data are mean ± s.d. (n = 3), results are representative of three independent experiments. $^{*}P < 0.05$ *versus* control. Statistical significance was determined using the Student's t-test.

a

Size (nm)	PDI	Zeta potential (mV)	EE (%)	DLE (%)
95.97 ± 0.22	0.176 ± 0.009	-23.2 ± 0.2	94.95 ± 0.34	2.35 ± 0.17

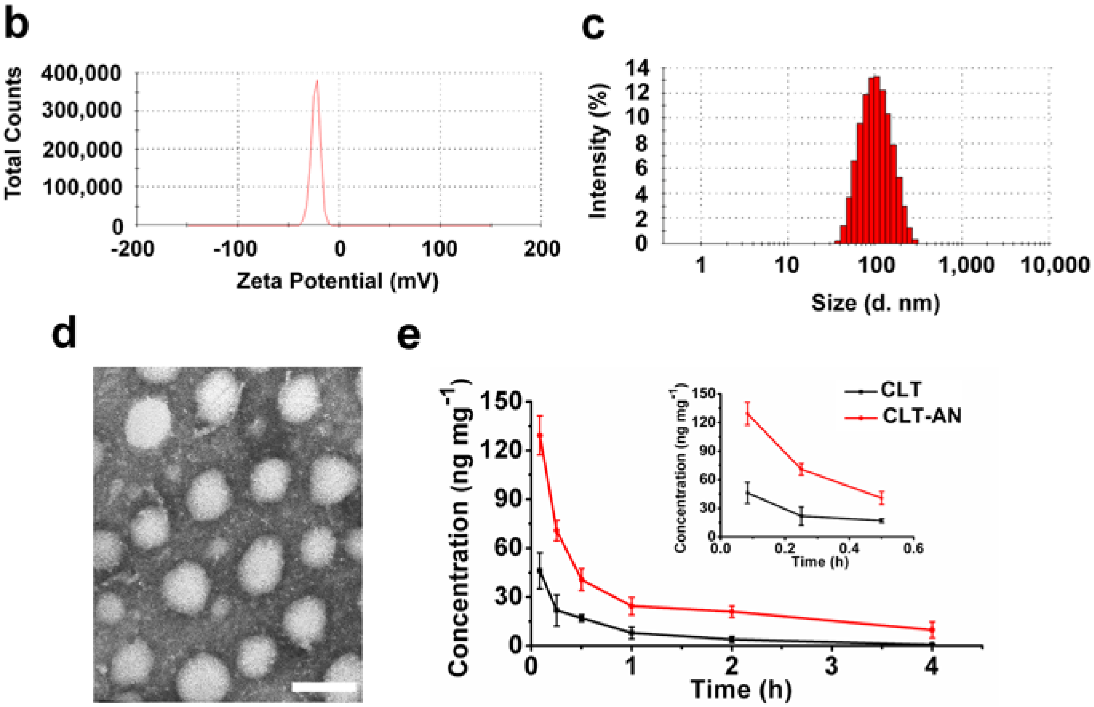

Supplementary Figure 13. Characterizations of CLT-AN and its specific glomeruli-targeted efficiency after intravenous administration into rats. (a) Size, polydispersity index (PDI), and zeta-potential of CLT-AN were measured using dynamic light scattering. Encapsulated and drug loading efficiency of CLT-AN were measured using high-performance liquid chromatography. Data are mean ± s.d. (*n* = 3), results are representative of three independent experiments. **(b)** Zeta potential of CLT-AN, as determined by dynamic light scattering. **(c)** Size distribution of CLT-AN, as determined by dynamic light scattering. **(d)** Transmission electron micrographs of CLT-AN. Scale bar, 100 nm. **(e)** Mean concentration-time curves of CLT and CLT-AN in glomeruli after intravenous administration into rats. Data are mean ± s.d. (*n* = 5), results are representative of two independent experiments.

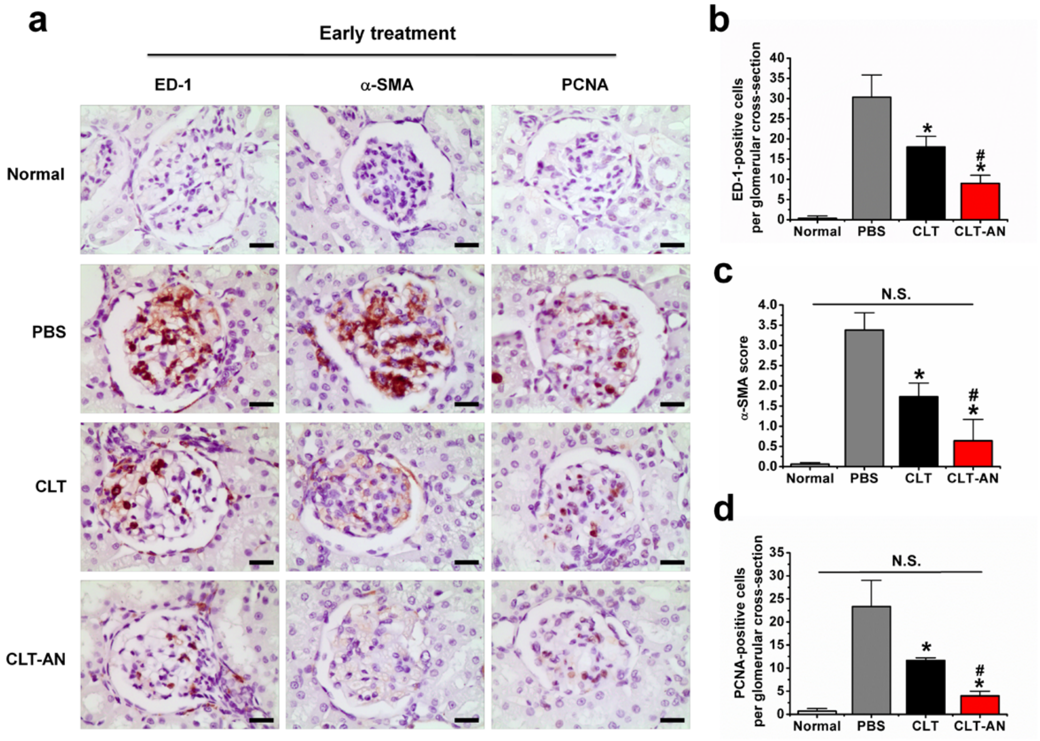

Supplementary Figure 14. The early CLT-AN treatment enhances inhibitory effects on macrophage infiltration, mesangial cell activation and proliferation in the reversible anti-Thy1.1 nephritis model. (a) Representative photomicrographs of immunostaining for ED-1, α-SMA and PCNA in kidney tissue sections from anti-Thy1.1 nephritic rats after early treatment with CLT and CLT-AN. Scale bars, 20 μm. **(b)** Effects of early CLT or CLT-AN treatment on macrophage infiltration on day 1 after disease induction. For each animal group, 150 glomeruli were selected and ED-1-positive cells were counted using cellSens Standard digital imaging software (Olympus). **(c)** Effects of early CLT or CLT-AN treatment on mesangial cell activation on day 5 after disease induction. For each animal group, 150 glomeruli were analyzed and α-SMA staining was graded semiquantitatively as described in Methods. **(d)** Effects of early CLT or CLT-AN treatment on mesangial cell proliferation on day 5 after disease induction. For each animal group, 150 glomeruli were selected and PCNA-positive cells were counted using cellSens Standard digital imaging software (Olympus). In panels **(b)** to **(d)**, data are mean ± s.d. (n = 5), results are representative of two independent experiments. N.S., not significant; $^{*}P < 0.05$ *versus* PBS group; $^{\#}P < 0.05$ *versus* CLT group. Statistical significance was determined by one-way ANOVA with Tukey *post hoc* test.

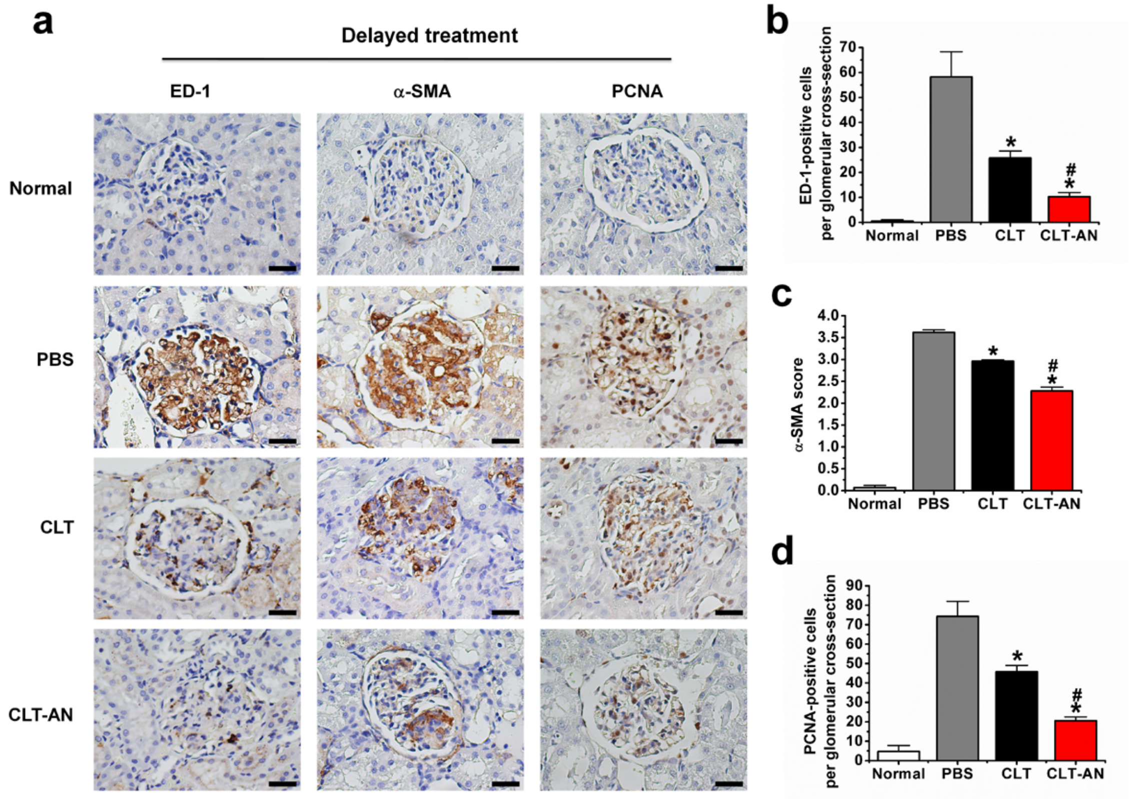

Supplementary Figure 15. The delayed CLT-AN treatment enhances inhibitory effects on macrophage infiltration, mesangial cell activation and proliferation in the reversible anti-Thy1.1 nephritis model. (a) Representative photomicrographs of immunostaining for ED-1, α-SMA and PCNA in kidney tissue sections from anti-Thy1.1 nephritic rats after delayed treatment with CLT and CLT-AN. Scale bars, 20 μm. **(b)** Effects of delayed CLT or CLT-AN treatment on macrophage infiltration on day 5 after disease induction. For each animal group, 150 glomeruli were selected and ED-1-positive cells were counted using cellSens Standard digital imaging software (Olympus). **(c)** Effects of delayed CLT or CLT-AN treatment on mesangial cell activation on day 5 after disease induction. For each animal group, 150 glomeruli were analyzed and α-SMA staining was graded semiquantitatively as described in Methods. **(d)** Effects of delayed CLT or CLT-AN treatment on mesangial cell proliferation on day 5 after disease induction. For each animal group, 150 glomeruli were selected and PCNA-positive cells were counted using cellSens Standard digital imaging software (Olympus). In panels **(b)** to **(d)**, data are mean ± s.d. (n = 5), results are representative of two independent experiments. $^{*}P < 0.05$ *versus* PBS group; $^{\#}P < 0.05$ *versus* CLT group. Statistical significance was determined by one-way ANOVA with Tukey *post hoc* test.

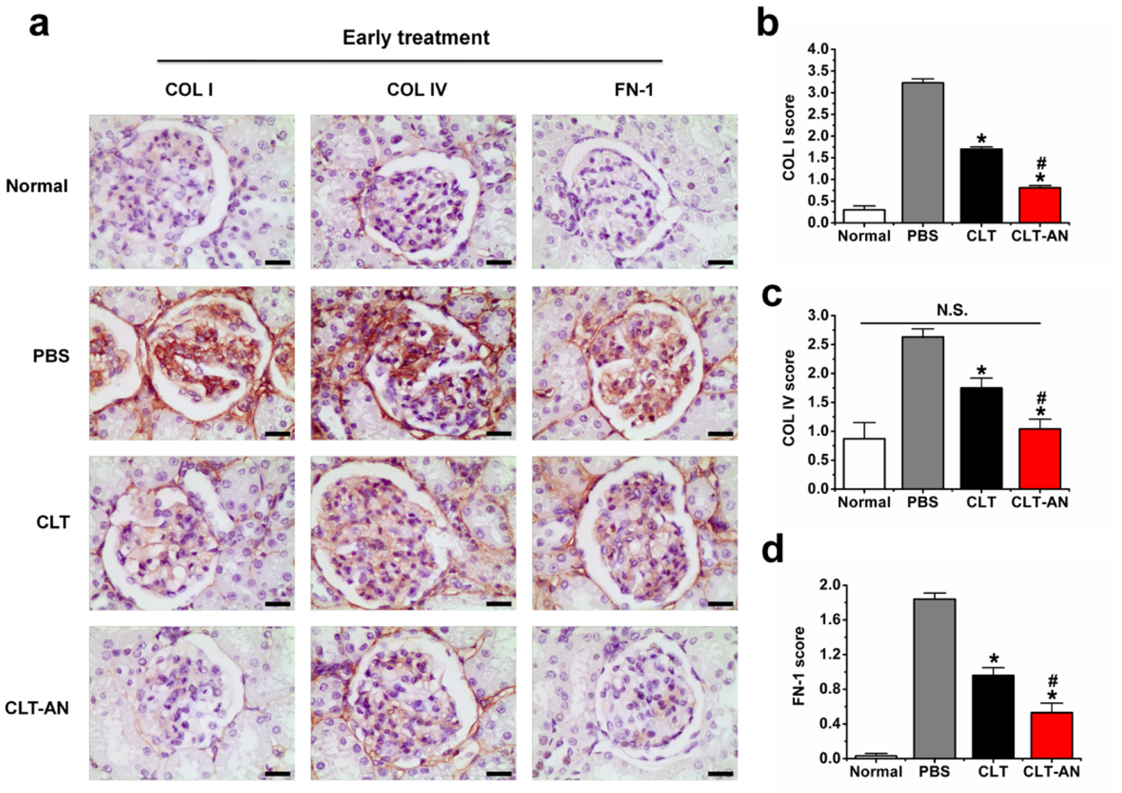

Supplementary Figure 16. The enhanced inhibitory effect of early therapy of CLT-AN on the deposition of ECM protein in the reversible anti-Thy1.1 nephritis model. (a) Representative photomicrographs of immunostaining for Col I, Col IV and FN-1 in kidney tissue sections from anti-Thy1.1 nephritic rats after early treatment with CLT or CLT-AN. Scale bars, 20 μm. **(b-d)** Effects of early CLT or CLT-AN treatment on deposition of Col I **(b)**, Col IV **(c)** and FN-1 **(d)** on day 5 after disease induction. For each animal group, 150 glomeruli were analyzed and staining of ECM proteins was graded semiquantitatively as described in Methods. In panels **(b)** to **(d)**, data are mean ± s.d. (n = 5), results are representative of two independent experiments. N.S., not significant; $^{*}P < 0.05$ *versus* PBS group; $^{\#}P < 0.05$ *versus* CLT group. Statistical significance was determined by one-way ANOVA with Tukey *post hoc* test.

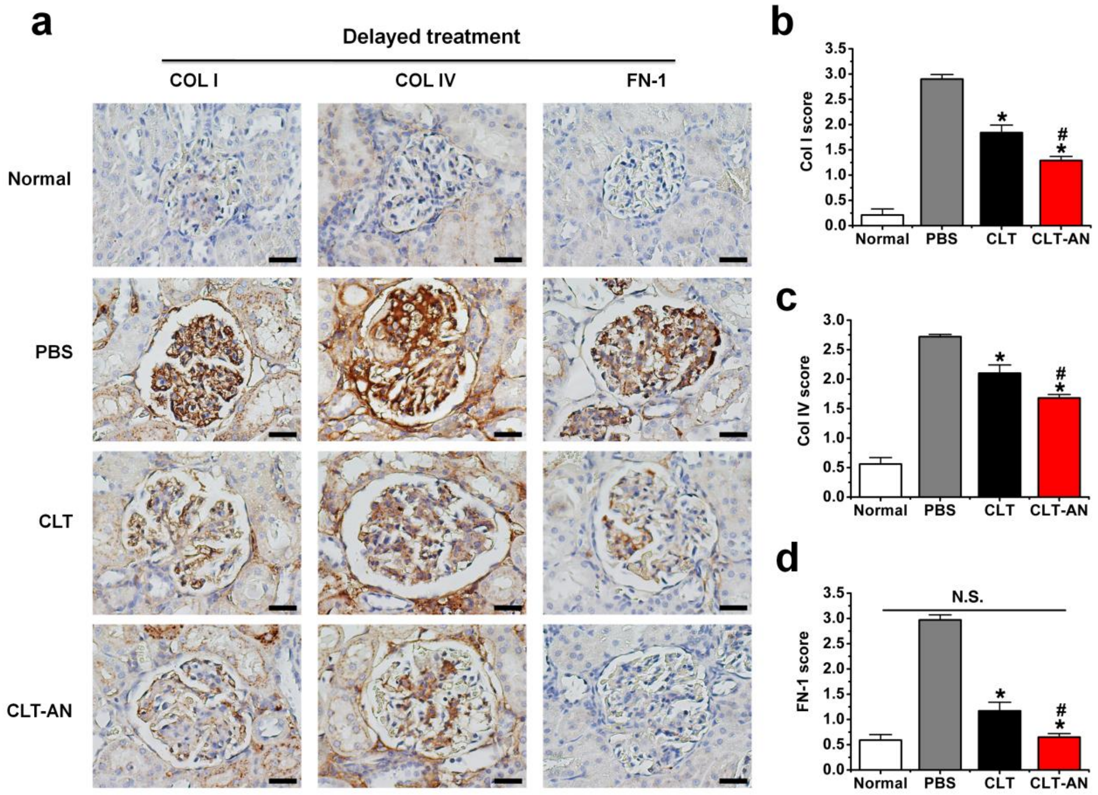

Supplementary Figure 17. The enhanced inhibitory effect of delayed therapy of CLT-AN on the deposition of ECM protein in the reversible anti-Thy1.1 nephritis model. (a) Representative photomicrographs of immunostaining for Col I, Col IV and FN-1 in kidney tissue sections from anti-Thy1.1 nephritic rats after delayed treatment with CLT or CLT-AN. Scale bars, 20 μm. **(b-d)** Effects of delayed CLT or CLT-AN treatment on deposition of Col I **(b)**, Col IV **(c)** and FN-1 **(d)** on day 5 after disease induction. For each animal group, 150 glomeruli were analyzed and staining of ECM proteins was graded semiquantitatively as described in Methods. In panels **(b)** to **(d)**, data are mean ± s.d. (n = 5), results are representative of two independent experiments. N.S., not significant; $^{*}P < 0.05$ *versus* PBS group; $^{\#}P < 0.05$ *versus* CLT group. Statistical significance was determined by one-way ANOVA with Tukey *post hoc* test.

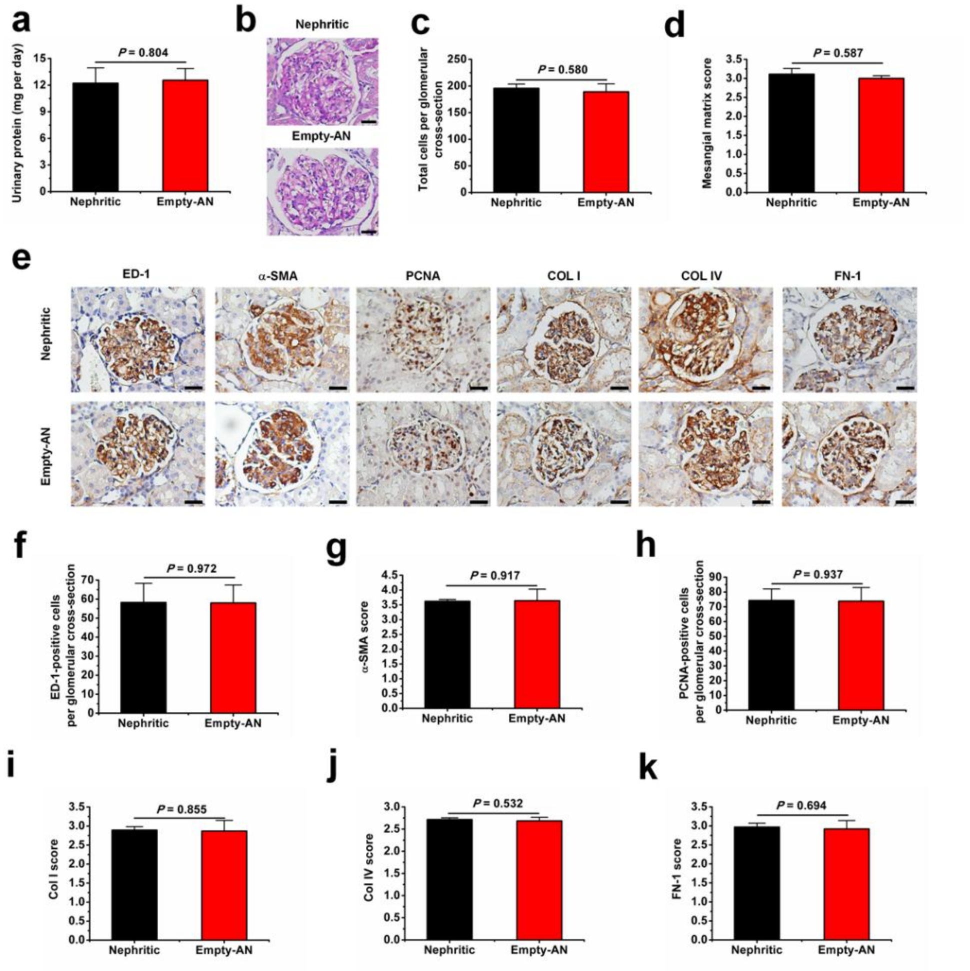

Supplementary Figure 18. Empty albumin nanoparticles (Empty-AN) treatment shows no significant benefits on proteinuria and glomerular lesions in the reversible anti-Thy1.1 nephritis model. (a) 24-h urinary protein excretion in anti-Thy1.1 nephritic rats on day 5 after disease induction. **(b)** Glomerular histology revealed by PAS staining of kidney tissue sections from anti-Thy1.1 nephritic rats on day 5 after treatment with empty-AN. Scale bars, 20μm. **(c)** The total glomerular cellularity on day 5 after disease induction. **(d)** The ECM deposition on day 5 after disease induction. **(e)** Representative photomicrographs of immunostaining for ED-1, α-SMA, PCNA, Col I, Col IV and FN-1 in kidney tissue sections from anti-Thy1.1 nephritic rats after delayed treatment with empty-AN. Scale bars, 20 μm. **(f-k)** The immunochemical results of ED-1 **(f)**, α-SMA **(g)**, PCNA **(h)**, Col I **(i)**, Col IV **(j)** and FN-1 **(k)** were semiquantitatively evaluated as described in Methods. In all panels, data are mean ± s.d. (n = 5), results are representative of two independent experiments. P values were calculated by Student's t-test.

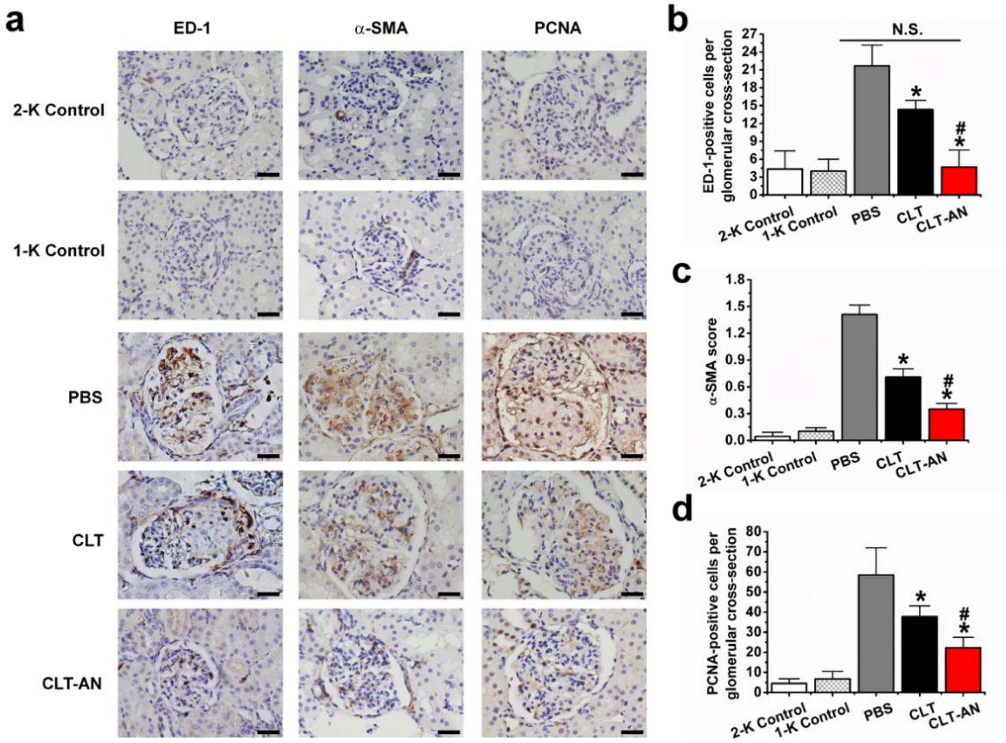

Supplementary Figure 19. The delayed CLT-AN treatment enhances inhibitory effects on macrophage infiltration, mesangial cell activation and proliferation in the irreversible anti-Thy1.1 nephritis model. (a) Representative photomicrographs of immunostaining for ED-1, α-SMA and PCNA in kidney tissue sections from anti-Thy1.1 nephritic rats after delayed treatment with CLT and CLT-AN. Scale bars, 20 μm. **(b)** Effects of delayed CLT or CLT-AN treatment on macrophage infiltration on day 14 after disease induction. For each animal group, 150 glomeruli were selected and ED-1-positive cells were counted using cellSens Standard digital imaging software (Olympus). **(c)** Effects of delayed CLT or CLT-AN treatment on mesangial cell activation on day 14 after disease induction. For each animal group, 150 glomeruli were analyzed and α-SMA staining was graded semiquantitatively as described in Methods. **(d)** Effects of delayed CLT or CLT-AN treatment on mesangial cell proliferation on day 14 after disease induction. For each animal group, 150 glomeruli were selected and PCNA-positive cells were counted using cellSens Standard digital imaging software (Olympus). In panels **(b)** to **(d)**, data are mean ± s.d. (n = 5), results are representative of two independent experiments. Nonnephrectomized two-kidney control (2-K Control) and uninephrectomized one-kidney control (1-K Control) served as controls. $^{*}P < 0.05$ *versus* PBS group; $^{\#}P < 0.05$ *versus* CLT group. Statistical significance was determined by one-way ANOVA with Tukey *post hoc* test.

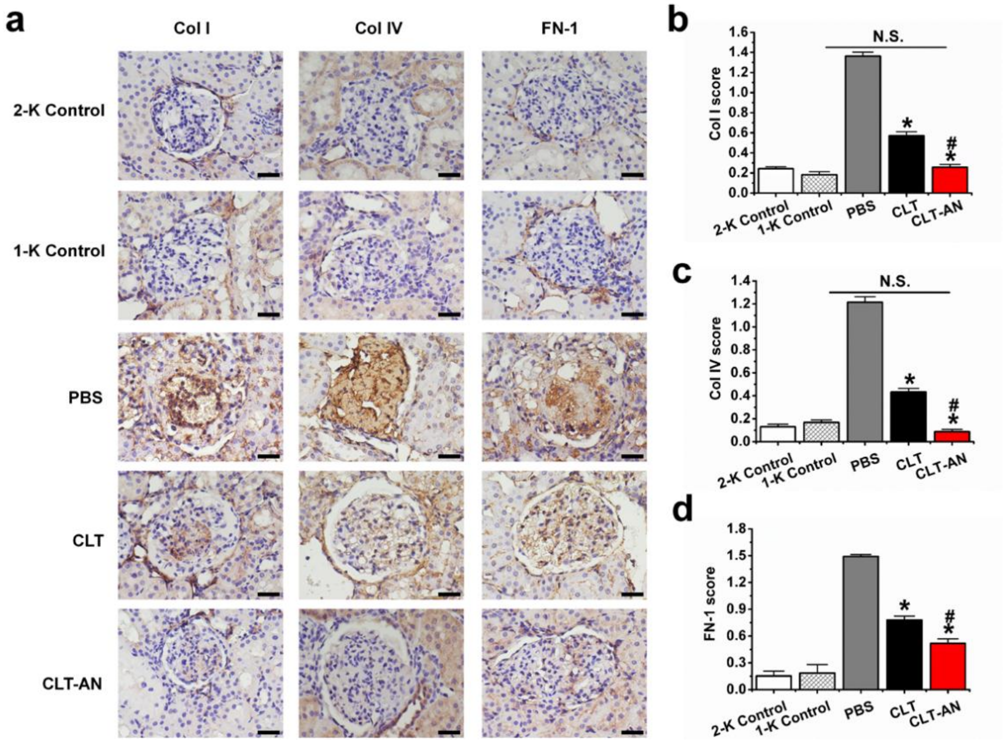

Supplementary Figure 20. The enhanced inhibitory effect of delayed therapy of CLT-AN on the deposition of ECM protein in the irreversible anti-Thy1.1 nephritis model. (a) Representative photomicrographs of immunostaining for Col I, Col IV and FN-1 in kidney tissue sections from anti-Thy1.1 nephritic rats after delayed treatment with CLT or CLT-AN. Scale bars, 20 μm. **(b-d)** Effects of delayed CLT or CLT-AN treatment on deposition of Col I **(b)**, Col IV **(c)** and FN-1 **(d)** on day 14 after disease induction. For each animal group, 150 glomeruli were analyzed and staining of ECM proteins was graded semiquantitatively as described in Methods. In panels **(b)** to **(d)**, data are mean ± s.d. (n = 5), results are representative of two independent experiments. Nonnephrectomized two-kidney control (2-K Control) and uninephrectomized one-kidney control (1-K Control) served as controls. N.S., not significant; $^{*}P < 0.05$ *versus* PBS group; $^{\#}P < 0.05$ *versus* CLT group. Statistical significance was determined by one-way ANOVA with Tukey *post hoc* test.

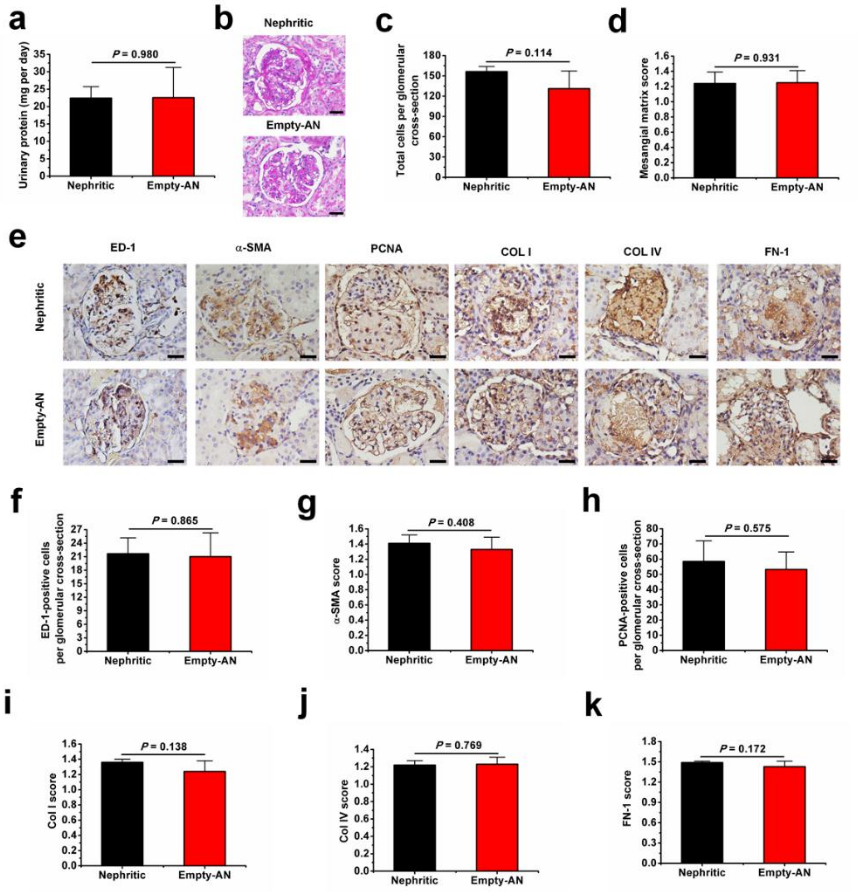

Supplementary Figure 21. Empty-AN treatment shows no significant benefits on proteinuria and glomerular lesions in the irreversible anti-Thy1.1 nephritis model. **(a)** 24-h urinary protein excretion in anti-Thy1.1 nephritic rats on day 14 after disease induction. **(b)** Glomerular histology revealed by PAS staining of kidney tissue sections from anti-Thy1.1 nephritic rats on day 14 after treatment with empty-AN. Scale bars, 20μm. **(c)** The total glomerular cellularity on day 14 after disease induction. **(d)** The ECM deposition on day 14 after disease induction. **(e)** Representative photomicrographs of immunostaining for ED-1, α-SMA, PCNA, Col I, Col IV and FN-1 in kidney tissue sections from anti-Thy1.1 nephritic rats after delayed treatment with empty-AN. Scale bars, 20 μm. **(f-k)** The immunochemical results of ED-1 **(f)**, α-SMA **(g)**, PCNA **(h)**, Col I **(i)**, Col IV **(j)** and FN-1 **(k)** were semiquantitatively evaluated as described in Methods. In all panels, data are mean ± s.d. (n = 5), results are representative of two independent experiments. P values were calculated by Student's t-test.

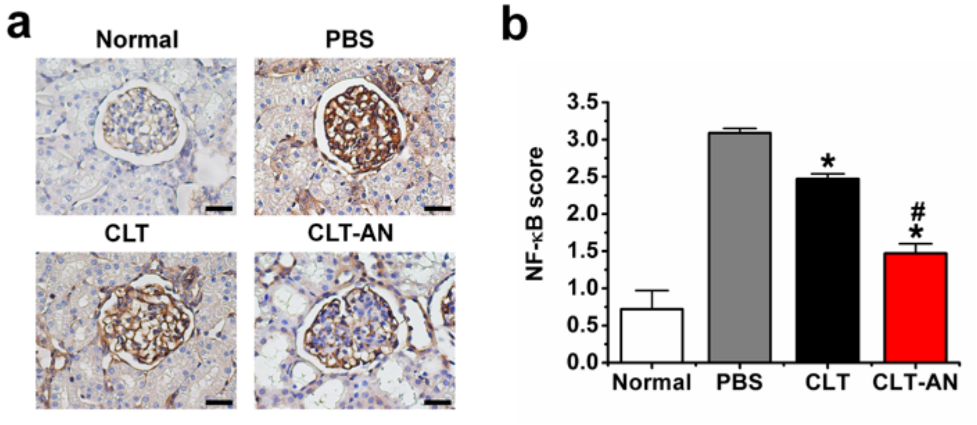

Supplementary Figure 22. The inhibitory effects of CLT and CLT-AN on the expression of NF-κB in the reversible anti-Thy1.1 nephritis model. (a) Representative photomicrographs of immunostaining for NF-κB in kidney tissue sections taken from anti-Thy1.1 nephritic rats on day 1 after treatment with CLT or CLT-AN. Scale bars, 20 μm. **(b)** The levels of NF-κB were semiquantitatively scored as described in Methods based on immunohistochemical results. Data are mean ± s.d. (n = 5), results are representative of two independent experiments. $^{*}P < 0.05$ *versus* PBS group; $^{\#}P < 0.05$ *versus* CLT group. Statistical significance was determined by one-way ANOVA with Tukey *post hoc* test.

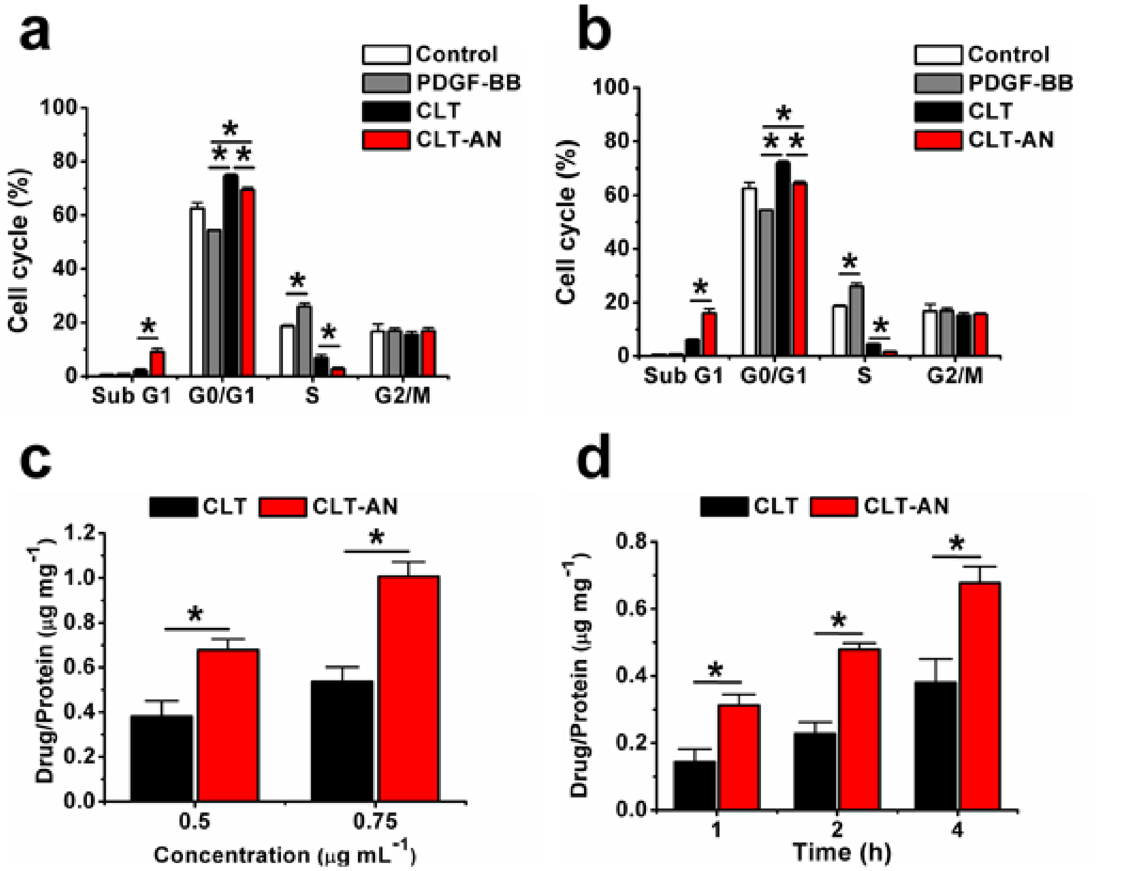

Supplementary Figure 23. ***In vitro*** **cellular evaluations of CLT and CLT-AN. (a-b)** Flow cytometry analysis of cell cycle distribution of HBZY-1 cells in the presence of PDGF-BB after exposure to CLT or CLT-AN at concentrations of 0.5 μg mL^{-1} **(a)** or 0.75 μg mL^{-1} **(b)**. In panels **(a)** and **(b)**, data are mean ± s.d. (n = 3), $^{*}P < 0.05$. Statistical significance was determined by one-way ANOVA with Tukey *post hoc* test. **(c)** Efficiency of CLT or CLT-AN uptake by HBZY-1 cells after 4-h exposure to different concentrations. **(d)** Efficiency of CLT or CLT-AN uptake by HBZY-1 cells after exposure to 0.5 μg mL^{-1} for 1, 2 or 4 h. In panels **(c)** and **(d)**, data are mean ± s.d. (n = 3), results are representative of three independent experiments. $^{*}P < 0.05$. Statistical significance was determined by Student's t-test.

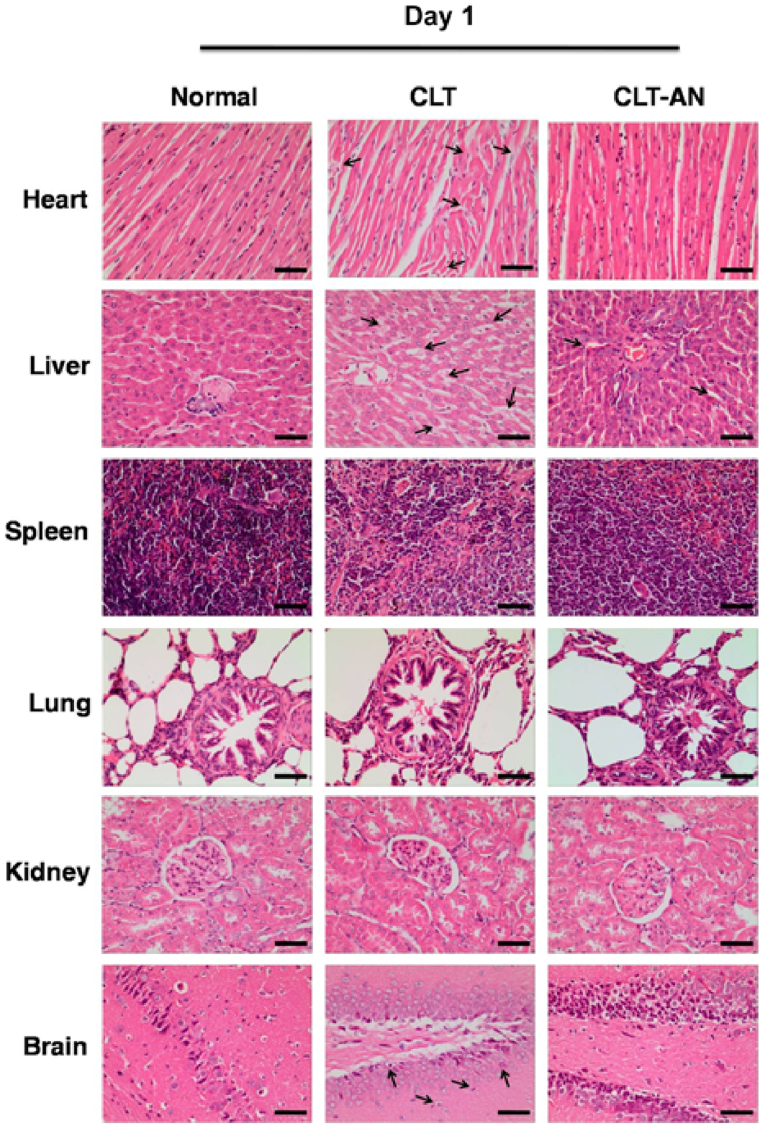

Supplementary Figure 24. Representative photomicrographs of heart, liver, spleen, lung, kidney and brain on day 1 after CLT or CLT-AN treatment. Tissue sections were isolated and stained with hematoxylin and eosin for histopathological analysis. Black arrows in heart samples indicate atrophy of myocardial cells and myofibrillar loss; arrows in liver samples indicate atrophy of hepatic cells and dilatation of blood sinus; arrows in brain samples show pyknosis of neuron and red neurons. Scale bars, 50 μm.

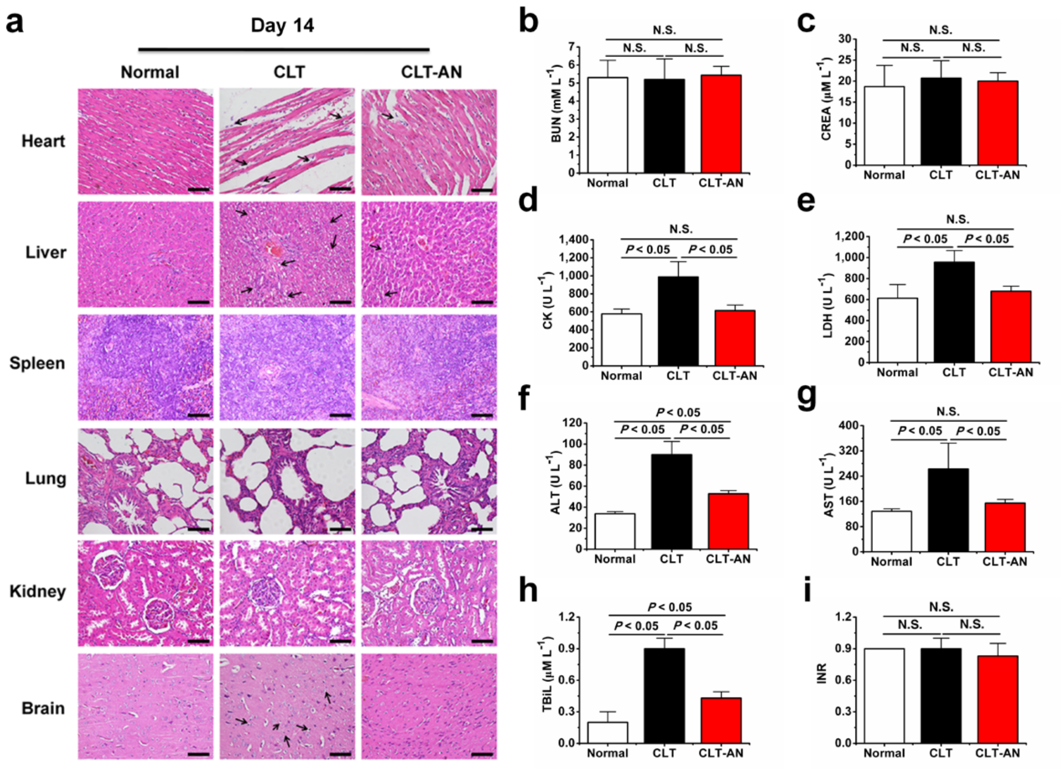

Supplementary Figure 25. CLT-AN reduces systemic toxicity of CLT in rats. (a) Hematoxylin & eosin (H&E) staining assay of heart, liver, spleen, lung, kidney and brain on day 14 after CLT or CLT-AN treatment. Black arrows in heart samples indicate atrophy of myocardial cells and myofibrillar loss; arrows in liver samples indicate the edema of hepatic cells; and arrows in brain samples indicate pyknosis of neuron and red neurons. Scale bars, 50 μm. **(b-i)** BUN, CREA, CK, LDH, ALT, AST, TBiL and INR levels on day 14 after CLT or CLT-AN treatment. Data are mean ± s.d. (n = 5), results are representative of two independent experiments. Statistical significance was determined by one-way ANOVA with Tukey *post hoc* test. N.S., not significant.

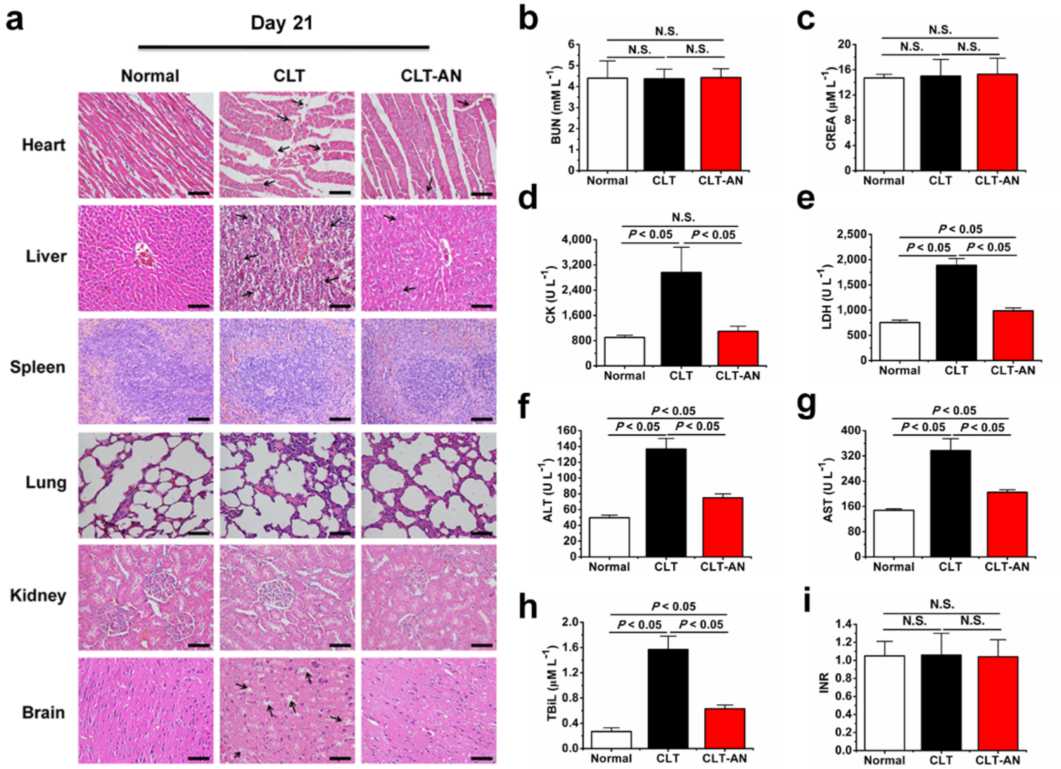

Supplementary Figure 26. CLT-AN reduces systemic toxicity of CLT in rats. (a) Hematoxylin & eosin (H&E) staining assay of heart, liver, spleen, lung, kidney and brain on day 21 after CLT or CLT-AN treatment. Black arrows in heart samples indicate atrophy of myocardial cells and myofibrillar loss; arrows in liver samples indicate the diffuse edema of hepatic cells; and arrows in brain samples indicate liquefactive necrosis foci. Scale bars, 50 μm. **(b-i)** BUN, CREA, CK, LDH, ALT, AST, TBiL and INR levels on day 21 after CLT or CLT-AN treatment. Data are mean ± s.d. (n = 5), results are representative of two independent experiments. Statistical significance was determined by one-way ANOVA with Tukey *post hoc* test. N.S., not significant.

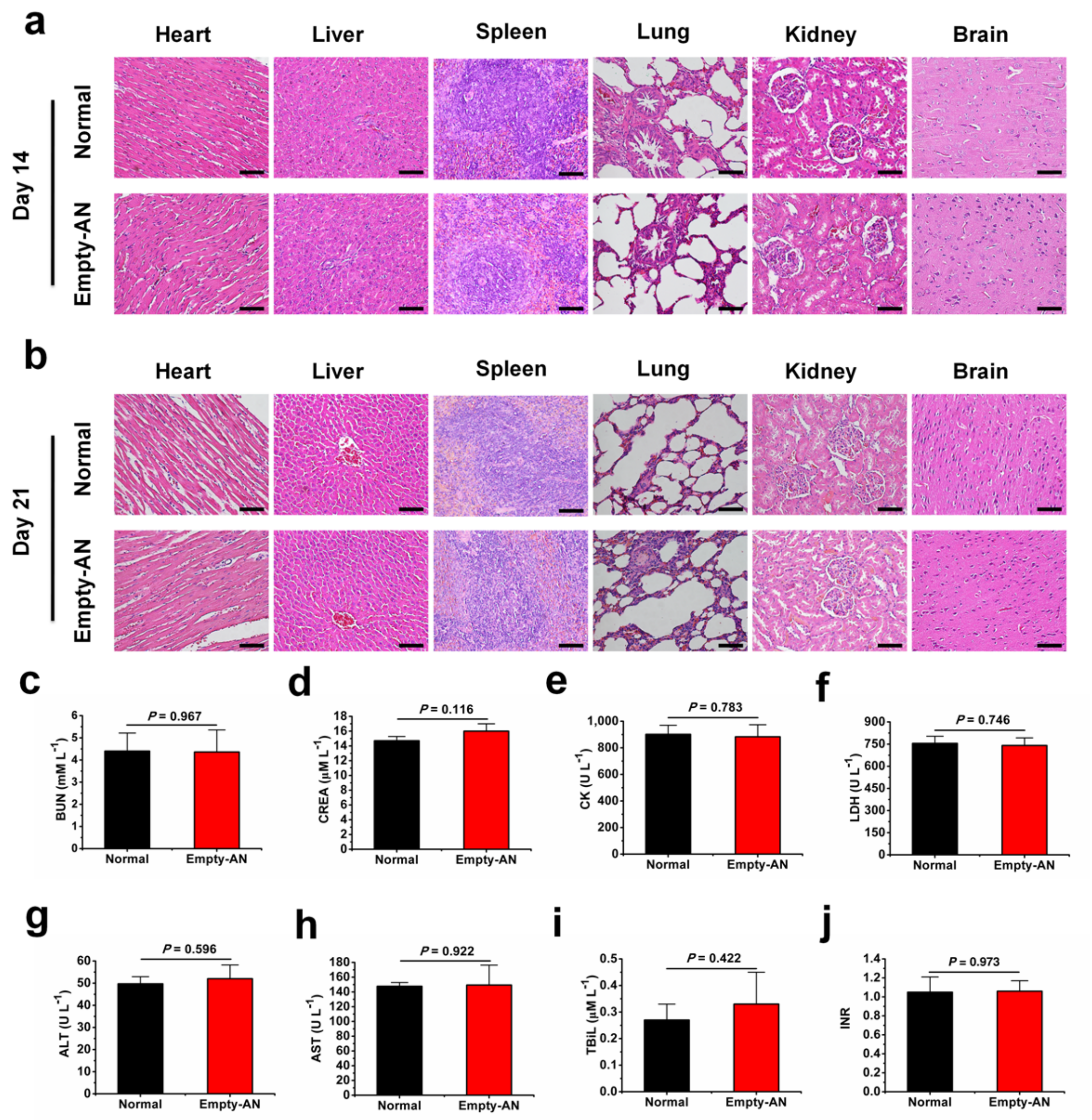

Supplementary Figure 27. No visible signs of systemic toxicity are observed in rats post exposure to empty-AN. (a-b) Hematoxylin & eosin (H&E) staining assay of heart, liver, spleen, lung, kidney and brain on day 14 **(a)** and day 21 **(b)** after empty-AN treatment. Scale bars, 50 μm. **(c-j)** BUN, CREA, CK, LDH, ALT, AST, TBiL and INR levels on day 21 after empty-AN treatment. Data are mean ± s.d. (n = 5), results are representative of two independent experiments. Statistical significance was determined by Student's t-test.

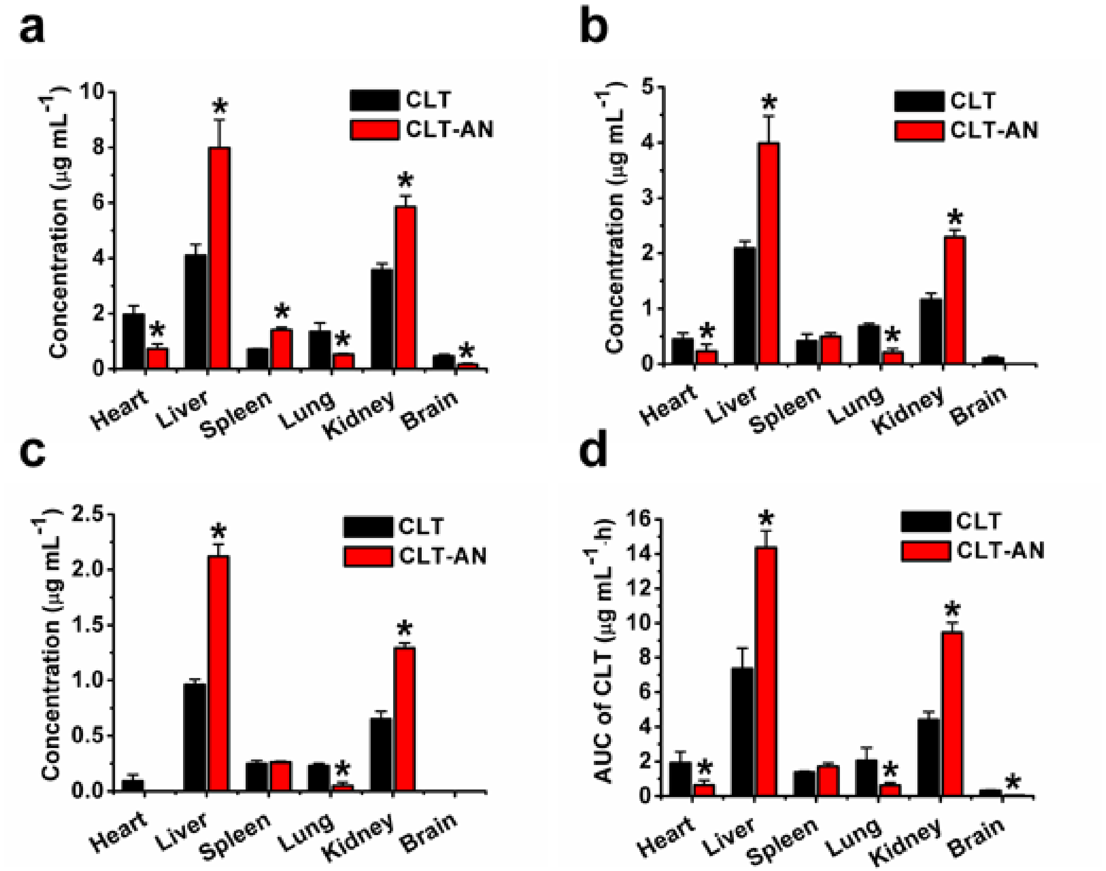

Supplementary Figure 28. Encapsulation of CLT in albumin nanoparticles (CLT-AN) alters its biodistribution in rats. (a-c) Tissue distribution in rats at 5 min **(a)**, 1 h **(b)**, and 4 h **(c)** after intravenous administration of CLT or CLT-AN. **(d)** $AUC_{0\text{–}4\,h}$ of CLT in different organs. In all panels, data are mean ± s.d. (*n* = 5), results are representative of two independent experiments. *$P < 0.05$, statistical significance was determined using the Student's *t*-test.

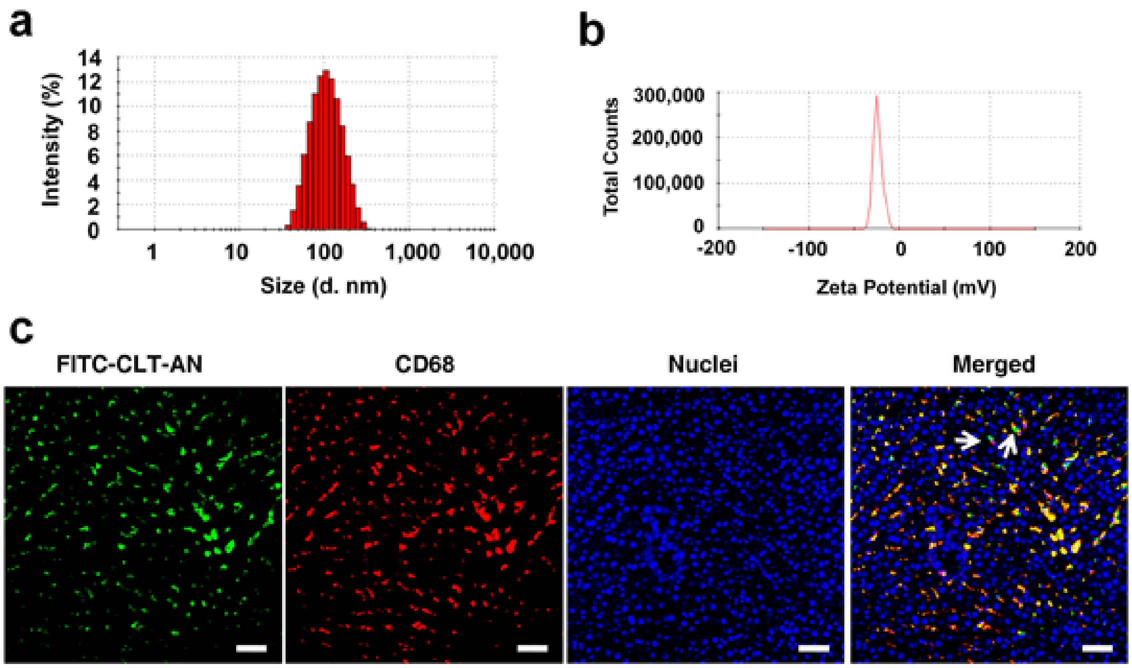

Supplementary Figure 29. CLT-AN are largely retained in CD68⁺ Kupffer cells in the liver. (a) Size distribution of FITC-CLT-AN, as determined by dynamic light scattering. **(b)** Zeta potential of FITC-CLT-AN, as determined by dynamic light scattering. **(c)** Representative confocal images of FITC-CLT-AN-treated liver tissue clearly showed that CLT-AN highly colocalize with $CD68^+$ Kupffer cells. Liver tissue sections obtained at 5 min after tail vein injection of FITC-CLT-AN (green) were counter-stained with Alexa Fluor®594 conjugated antibody against the macrophage marker CD68 (red). Blue denotes DAPI stain for cell nuclei, and Kupffer cells retaining CLT-AN are shown in yellow in the merged images. The white arrows indicate a few CLT-AN-positive, but CD68-negative cells. Scale bars, 50 µm.

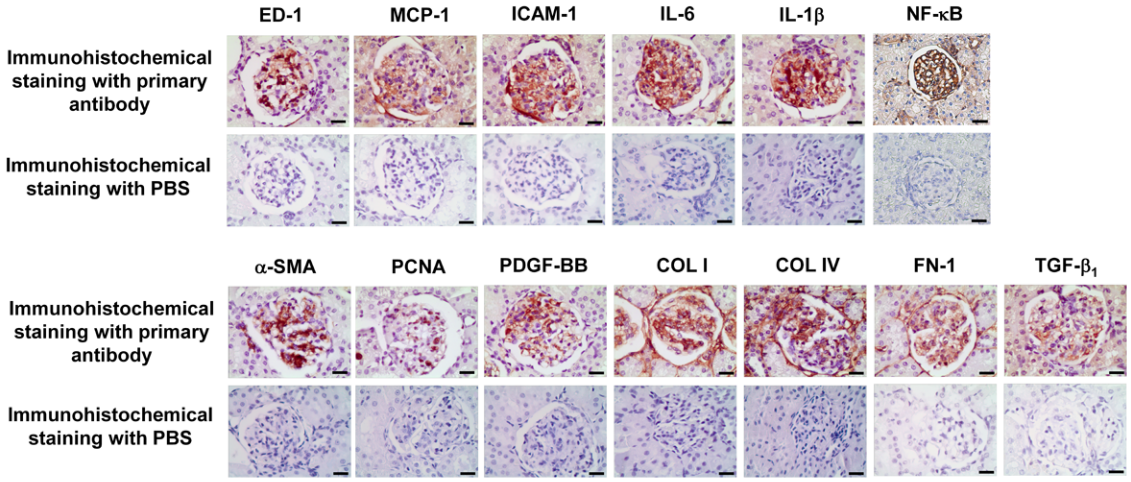

Supplementary Figure 30. No staining is observed in kidney tissues sections when the primary antibody was replaced with PBS in immunohistochemical analysis. The kidney tissue sections taken from anti-Thy1.1 nephritic rats on day 1 or 5 after disease induction were immunostained for ED-1, MCP-1, ICAM-1, IL-6, IL-1, NF-κB, α-SMA, PCNA, PDGF-BB, Col I, Col IV, FN-1, TGF-β_1 with the corresponding primary antibody or PBS. Scale bars, 20 μm.

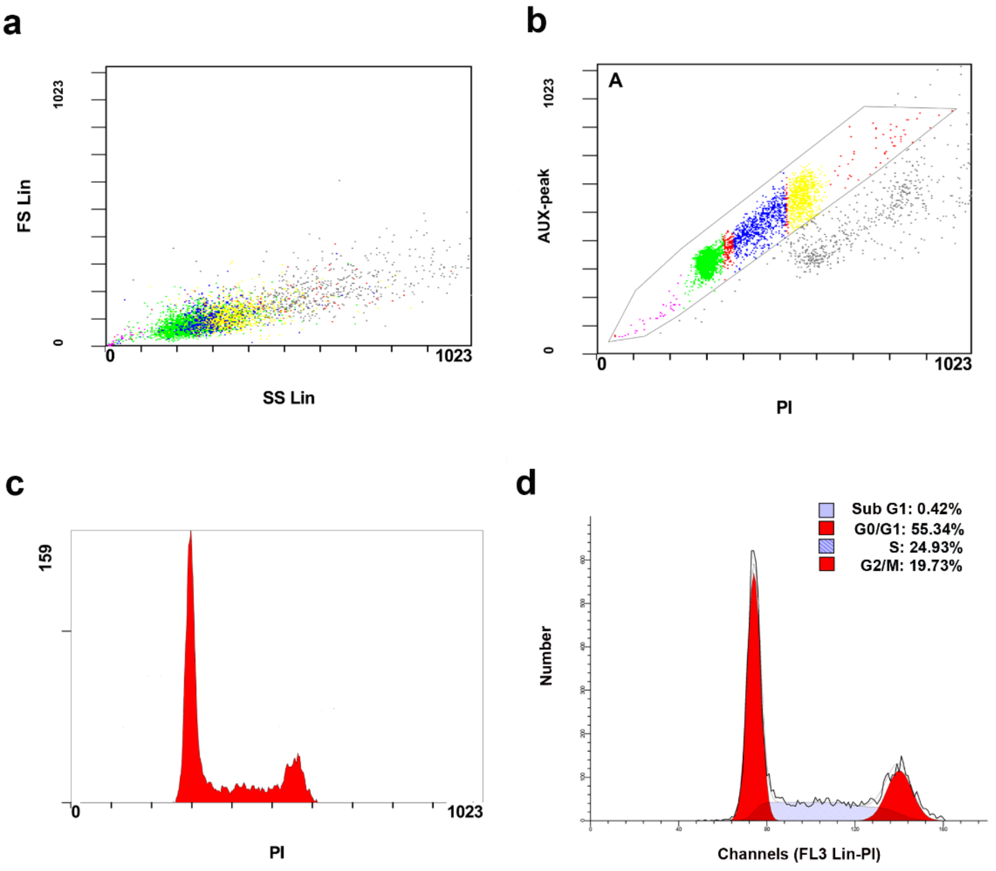

Supplementary Figure 31. Flow cytometry gating strategy for the cell cycle assay. Representative data obtained from flow-cytometric analysis of cell cycle of HBZY-1 cells treated with PDGF-BB**.** Cells were stained with the DNA binding dye PI and subjected to flow cytometry as described in Methods section. First, the main cell population was gated using a forward/side scatter (FS/SS) dot plot **(a)**, and a AUX-peak/PI plot **(b)** was used to gate the single cell**.** This gate applied to the PI plot **(c)** was also used to exclude aggregates. The population histogram for the sample was then obtained, and the percentage of HBZY-1 cells in Sub G1, G0/G1, S, G2/M phases of the cell cycle can be calculated using the ModFit LT 2.0 software **(d)**.

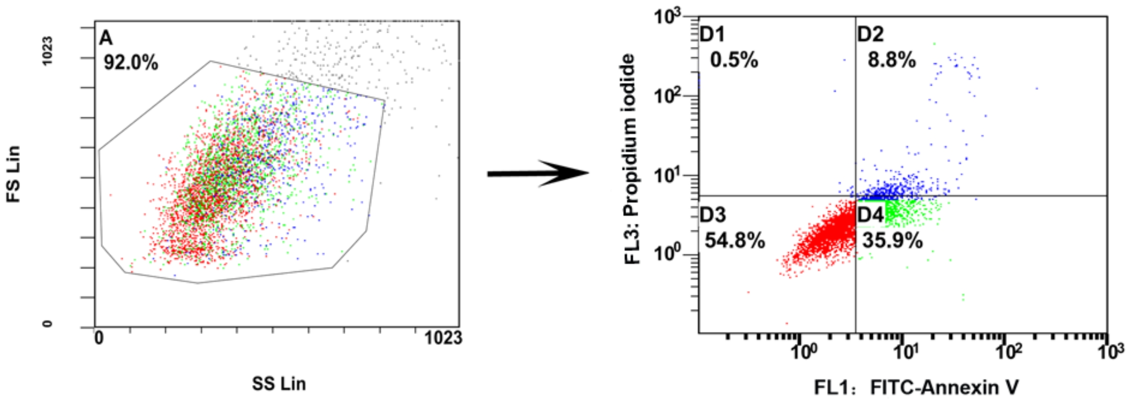

Supplementary Figure 32. Flow cytometry gating strategy for the cell apoptosis assay. A forward/side scatter (FS/SS) dot plot (left) was used to gate the main cell population, and the gated cells were then analyzed by FITC-Annexin V and PI staining patterns in a FL1/FL2 plot (right). the proportion of cells undergoing viable (double-negative for FITC-annexin V and PI), early apoptosis (positive for FITC-annexin V), late apoptosis (double-positive for FITC- annexin V and PI), and necrosis (positive for PI) can be found in quadrants D3, D4, D2 and D1, respectively. In our study, the total percentage of apoptosis induction is defined as the sum of early apoptotic and late apoptotic cells.

Supplementary Table 1. Pharmacokinetic parameters of CLT and CLT-AN in glomeruli after intravenous administration into rats (*n* = 5)

Parameters	CLT	CLT-AN
$AUC_{0\text{-}t}$ (ng mg^{-1} h)	31.81 ± 4.70	112.85 ± 9.90 *
C_{max} (ng mg^{-1})	46.13 ± 10.98	129.27 ± 11.90 *
$MRT_{0\text{-}t}$ (h)	0.78 ± 0.11	1.18 ± 0.09 *
R_e	-	3.55
C_e	-	2.80

$AUC_{0\text{–}t}$: area under drug concentration-time curve; C_{max}: maximum drug concentration in glomeruli; $MRT_{0\text{-}t}$: mean residence time. R_e: relative uptake efficiency; C_e: concentration efficiency. Data are mean ± s.d. (n = 5), $^{*}P < 0.05$ *versus* CLT group.

Supplementary Table 2. Pharmacokinetic parameters of CLT and CLT-AN in major tissues after intravenous administration into rats (*n* = 5)

Parameters		$AUC_{(0\text{-}t)}$ (μg mL^{-1}·h)	C_{max} (μg mL^{-1})	$MRT_{0\text{-}t}$ (h)
Heart	CLT	1.92 ± 0.65	1.97 ± 0.31	1.04 ± 0.08
	CLT-AN	0.64 ± 0.27	0.73 ± 0.17	0.82 ± 0.13
Liver	CLT	7.36 ± 1.18	4.09 ± 0.41	1.49 ± 0.16
	CLT-AN	14.37 ± 0.97	7.99 ± 1.01	1.52 ± 0.11
Spleen	CLT	1.38 ± 0.06	0.71 ± 0.03	1.64 ± 0.12
	CLT-AN	1.73 ± 0.18	1.41 ± 0.09	1.46 ± 0.09
Lung	CLT	2.07 ± 0.73	1.35 ± 0.31	1.34 ± 0.15
	CLT-AN	0.63 ± 0.15	0.53 ± 0.03	1.20 ± 0.13
kidney	CLT	4.41±0.46	3.58 ± 0.22	1.44 ± 0.10
	CLT-AN	9.46 ± 0.57	5.85 ± 0.41	1.53 ± 0.13
Brain	CLT	0.32 ± 0.03	0.47 ± 0.06	0.61 ± 0.09
	CLT-AN	0.04 ± 0.02	0.17 ± 0.04	0.09 ± 0.03

$AUC_{0\text{–}t}$: area under drug concentration-time curve; C_{max}: maximum drug concentration; $MRT_{0\text{-}t}$: mean residence time.

Supplementary Table 3. The optimum conditions and sequences of primers used for quantitative RT-PCR gene expression studies

Description	Gene bank	Sequence of primer (5’-3’)<br>Antisense primer (3’-5’)	Anneal temperature (℃)	Cycles
MCP-1	NM_24770	CAGGTCTCTGTCACGCTTCT<br>GTAGTTCTCCAGCCGACTCA	64.5	40
ICAM-1	NM_25464	CTGTCGGTGCTCAGGTATCC<br>GTCTTCCCCAATGTCGCTCA	62.5	40
IL-6	NM_24498	CCAGTTGCCTTCTTGGGACT<br>GGTCTGTTGTGGGTGGTATCC	64.5	40
IL-1β	NM_24494	GCTACCTATGTCTTGCCCGT<br>TCACACACTAGCAGGTCGTC	64.5	40
PDGF-BB	NM_24628	CGTCTGTCTCGATGCCTGAT<br>TCTTGGGGTAGAGGGAAGCA	60.0	40
Col I	NM_29393	GCAATGCTGAATCGTCCCAC<br>CAGCACAGGCCCTCAAAAAC	59.9	40
Col IV	NM_290905	AGACAACAGATGACCCGCTG<br>GCGAAGTTGCAGACGTTGTT	57.1	40
FN-1	NM_25661	AACTTCTGGTCCTCTCCCGT<br>GGACCCCTGAGCATCTTGAG	56.8	40
TGF-β_1	NM_59086	CACTCCCGTGGCTTCTAGTG<br>CTGGCGAGCCTTAGTTTGGA	64.5	40
β-actin	NM_007393.3	CAGGTCCAGACGCAGGATGGC<br>CTACAATGAGCTGCGTGTGG	60	40

RT-PCR: Quantitative Real-time Polymerase Chain Reaction.
